# Screening Study on the Anti-Angiogenic Effects of Traditional Chinese Medicine - Part II: *Wild Chrysanthemum*

**DOI:** 10.7150/jca.52971

**Published:** 2021-01-01

**Authors:** Xiang Tu, He Bin Wang, Qun Huang, Yi Cai, Yuan Ping Deng, Zhe Yong, Quan Hu, Jian Feng, James B. Jordan, Sen Zhong

**Affiliations:** 1 Cancer Research Institute, Hospital of Chengdu University of Traditional Chinese Medicine, Chengdu 610072, Sichuan Province, China; 2 Department of Hepatology, Affiliated Hospital of Panzhihua University, Panzhihua 617000, Sichuan Province, China; 3 Department of Pathology, 363 Hospital of Aviation Industry Corporation of China, Ltd., Chengdu 610041, Sichuan Province, China; 4 Department of Oncology, Hospital of Chengdu University of Traditional Chinese Medicine, Chengdu 610072, Sichuan Province, China; 5 Department of Internal Medicine, Traditional Chinese Medicine Hospital of Fushun County, Fushun 643200, Sichuan Province, China; 6 Department of Acupuncture & Moxibustion, Sichuan Second Traditional Chinese Medicine Hospital, Chengdu 610031, Sichuan Province, China; 7 Department of Gerontology, Hospital of Chengdu University of Traditional Chinese Medicine, Chengdu 610072, Sichuan Province, China; 8 Sichuan Neo-Green Pharmaceutical Technology Development Co., Ltd., Pengzhou 611930, Sichuan Province, China

**Keywords:** *wild chrysanthemum*, zebrafish, angiogenesis, proteasome, β-catenin, Traditional Chinese Medicine

## Abstract

**Background and Aims:** Part 2 of our ongoing research with anti-angiogenic effects focuses on *Wild chrysanthemum;* a *heat-clearing and detoxicating* Traditional Chinese Medicine (TCM). We screened six *heat-clearing and detoxicating* TCM and noticed that *wild chrysanthemum* has a potent anti-angiogenic effect in zebrafish. This study aims to determine the genetic mechanisms underlying the anti-angiogenic effects of *wild chrysanthemum*.

**Methods:** *Wild chrysanthemum* was decocted, concentrated, sieved and desiccated to attain the water extract. 200μg/mL *wild chrysanthemum* water extract (WCWE) was diluted in 0.1% dimethyl sulfoxide (DMSO) and given to zebrafish via fish water. 48h post-fertilization (hpf) *fli1a-EGFP* transgenic zebrafish were used to assay angiogenesis. mRNA-seq, qRT-PCR assay and a parallel reaction monitor (PRM) were carried out to reveal the underlying mechanisms.

**Results:** WCWE showed a significant anti-angiogenic effect in zebrafish. The results of mRNA-seq showed that there were 1119 genes up-regulated and 1332 genes down-regulated by WCWE. The bioinformatic analysis based on mRNA-seq demonstrated that the proteasome signaling pathway was significantly down-regulated. The results of the qRT-PCR assay were consistent with those of the mRNA-seq assay. The results of the PRM assay showed that nine proteins involved in proteasome signaling and the protein expression level of ctnnb2 were significantly down-regulated. The results of the KEGG pathway analysis based on PRM assay demonstrated that WCWE may have an inhibitory action on the regulatory particle of the proteasome.

**Conclusion:** *Wild chrysanthemum* has a significant anti-angiogenic effect in zebrafish and it may have an inhibitory action on the regulatory particle of the proteasome. The mechanisms underlying the anti-angiogenic effects of *wild chrysanthemum* may be related to the down-regulation of proteasome/β-catenin signaling in zebrafish.

## Introduction

*Wild chrysanthemum* was first recorded as a Traditional Chinese Medicine (TCM) in *Shennong's Classic of Materia Medica* (anonymous, Han dynasty). During the Ming and Qing dynasties, it was primarily applied externally for the management of abscesses or deep-rooted boils. The clinical applications have greatly expanded over thousands of years. It is widely accepted that the major actions of *Wild chrysanthemum* are *heat-clearing* and* detoxicating*; *fire-purging* and* liver-pacifying* 1. In modern TCM textbooks, *Wild chrysanthemum* is classified as a *Heat-clearing and detoxicating* TCM - the most frequently used category in the treatment of cancerous tumors 2. With the development of modern TCM pharmacology, Chinese experts discovered that *Wild chrysanthemum* contains many flavonoid compounds; and total flavonoids Chrysanthemum (TFC) are the major ingredients of *Wild chrysanthemum* 3. Herbal flavonoids have attracted the attention of the cancer research community and a number of literature report that TFC has anti-cancer potency 4.

We screened six *heat-clearing* and* detoxicating* Traditional Chinese Medicine (TCM) and noticed that *wild chrysanthemum* has potent anti-angiogenic effects in zebrafish 5. However, it remains unclear which signaling was regulated by *wild chrysanthemum*. This study aims to fully determine the mechanisms underlying the anti-angiogenic effect of* wild chrysanthemum*.

## Materials and Methods

### *Wild chrysanthemum* water extract (WCWE)

*Wild chrysanthemum* (Pinyin name: Ye Ju Hua; Latin name: *Chrysanthemum indicum* L.) was purchased from Si-Chuan Rejuvenation Hall Pharmaceutical Co., LTD. and produced in Hebei Province, China. The quality of *wild chrysanthemum* (batch number: 150301) complies with the *Pharmacopoeia of China* (2015 edition). Professor HouLin Xia from the College Pharmacy, Chengdu University of TCM, confirmed that *wild chrysanthemum* (plant parts used: capitulum) was the right herbal species recorded in the *Pharmacopoeia of China* (2015 Edition) 1. Suining (Sichuan, China) FDA assayed the quality standards of the *wild chrysanthemum* and provided confirmation quality reports that the standards meet the Chinese Pharmacopeia requirements. The *wild chrysanthemum* water extract (WCWE) was prepared as described in our previous published article 5. The extraction methods including extraction solvent, extraction conditions etc. were reported in Tu et al., 2016 5. The extract ratio of *wild chrysanthemum* was 37.3%. WCWE was diluted in 0.1% dimethyl sulfoxide (DMSO, Cat# D2650, Sigma).

### Zebrafish angiogenesis assay

22 h post-fertilization (hpf) *fli1a-EGFP* transgenic zebrafish were used to assay angiogenesis *in vivo*. In short, 200μg/mL WCWE was given to the larvae via fish water for 26 h and the larvae at 48 hpf were used for the angiogenesis assay. The positive control was 5μg/ml PTK 787 (Vatalanib, Selleckchem, USA, batch number: S110102). The vehicle control was 0.1% DMSO (comparable to fish water). Zebrafish angiogenesis assay details were the same as we described in our previous published article 5 and the experiments are repeated here. The ethics committee of the Teaching Hospital of Chengdu University of TCM approved all experiments and the zebrafish care complied with the ARRIVE guidelines.

### Sample preparation and mRNA‑Seq

The zebrafish received WCWE for the angiogenesis assay at 48 hpf, then homogenized; and the homogenates were used for mRNA Seq. The mRNA Seq was completed by the Shanghai KangChen Bio-tech Company, Shanghai, China. RNA was extracted by using Trizol kits (Invitrogen, USA). Extracted total RNA was characterized and quantified with agarose electrophoresis and a Nanodrop ND-1000 spectrophotometer (Thermo Fisher Scientific, Waltham, MA, USA). The mRNA was enriched by oligo(dT) and the rRNA was removed. Libraries were completed with the KAPA Stranded RNA-Seq Library Prep Kit Illumina (KK8400, USA, San Diego), which included a randomly primed 1st strand cDNA synthesis and a dUTP based 2nd strand cDNA synthesis. The sequencing library was determined by an Agilent 2100 Bioanalyzer (G2938C, Agilent USA Santa Clara) and quantified with a qPCR assay. A mRNA‑Seq was conducted with an Illumina Hiseq 4000 (Illumina Hiseq USA, San Diego).

### Quantitative real-time PCR (qRT-PCR) assay

The total RNA of zebrafish homogenates were extracted with a Trizol reagent and reverse transcribed with SuperScript III according to the manufacturer's instructions. A qRT-PCR was performed using a ViiA 7 Real-time PCR System. GAPDH was used as an internal control. Each PCR reaction mixture contained MgSO4, dNTP mixture, taq, and SYBR green. The primers used to amplify the genes were as follows:

β-actin zebrafish (F: 5' TGGCTTCTGCTCTGTATGGC 3'; R: 5' CCCTGTTAGACAACTACCTCCCT 3'), psme3 (F: 5' AACACAGTCAAGATGTGGGTT 3'; R: 5' CGGCGATAATCCTCAACA 3'), psmc6 (F:5' GCAACTAATGGACCACGAT 3' R: 5' CAGCCTTTGGGAGGAATA 3'), psmc3 (F: 5' TTTCTTGCCTGTGATTGG 3'; R: 5' AGTCATACTCGGTGGGTAGA 3'), psmc2 (F: 5' GGAGAAAGAGGACAAACCCAT 3'; R: 5' GCCAAATCCCATAGTGCC 3'), si:rp71-45k5.4 (F: 5' TTGAACCGATAACCAAACAC 3'; R: 5'GCTCTTGATAGACCAGGAAATAC3'), psmd4a (F: 5' TCAAAGACCCGCAGCAAC 3'; R: 5' TCTTGTGGTTCTTGCCTTGTC 3'), psmd8 (F: 5' GAGGGCAGTTACAACAAGGT 3'; R: 5' TCTCGGATGGTATCAAGAAGA 3'), psmd11a (F: 5' CAAGCCAGCAAGAATAGGTC 3'; R: 5' TTGTTCCAGCAGATTGTCATA 3'), psme4b (F: 5' TATCGGCAGCGTATTGAC 3'; R: 5' AGTGGGCAGAGTGTAGGG 3'), psma3 (F: 5' GAGTCTGCGGCTGCTAAT 3'; R: 5' AAAGTGGAGGCGGATAAA 3').

### Protein Extraction for the Parallel reaction monitor (PRM) assay

The sample was ground by liquid nitrogen into cell powder and then transferred to a 5-mL centrifuge tube. After that, four volumes of lysis buffer were added to the cell powder, followed by sonication three times on ice using a high intensity ultrasonic processor (Scientz). The lysis buffer contained: 8 M urea (Sigma, Saint Louis, USA, item number: V900119-500G); 1% Triton-100; 10 mM dithiothreitol (Sigma, item number: D9163-5G); and a 1% Protease Inhibitor Cocktail (Calbiochem, Billerica, USA, item number: 535140-1ML). The remaining debris were removed by centrifugation at 20,000 g at 4 °C for 10 min. Finally, the protein was precipitated with cold 20% TCA for 2 h at -20 °C. After centrifugation at 12,000 g 4 °C for 10 min, the supernatant was discarded. The remaining precipitate was washed with cold acetone for three times. The protein was redissolved in 8 M urea and the protein concentration was determined with a BCA kit according to the manufacturer's instructions.

### Trypsin Digestion

For digestion, the protein solution was reduced with 5 mM dithiothreitol for 30 min at 56 °C and alkylated with 11 mM iodoacetamide (Sigma, item number: V900335-5G) for 15 min at room temperature in darkness. The protein sample was then diluted to a urea concentration less than 2M. Finally, trypsin (Promega, Madison, USA. Item number: V5111) was added at 1:50 trypsin-to-protein mass ratio for the first digestion overnight and 1:100 trypsin-to-protein mass ratio for a second 4 h-digestion.

### LC-MS/MS Analysis

The tryptic peptides were dissolved in 0.1% formic acid (solvent A, Fluka, Saint Louis, USA, item number: 56302-50ML-F), directly loaded onto a home-made reversed-phase analytical column. The gradient was comprised of an increase from 6% to 23% solvent B (0.1% formic acid in 98% acetonitrile, Fisher Chemical, Waltham, USA, item number: A998-4) over 38 min, 23% to 35% in 14 min and climbing to 80% in 4 min then holding at 80% for the last 4 min; all at a constant flow rate of 700 nL/min on an EASY-nLC 1000 UPLC system.

The peptides were subjected to NSI source followed by tandem mass spectrometry (MS/MS) in Q ExactiveTM Plus (Thermo) coupled online to the UPLC. The electrospray voltage applied was 2.0 kV. The m/z scan range was 350 to 1000 for full scan, and intact peptides were detected in the Orbitrap at a resolution of 35,000. Peptides were then selected for MS/MS using NCE setting at 27 and the fragments were detected in the Orbitrap at a resolution of 17,500. A data-independent procedure that alternated between one MS scan followed by 20 MS/MS scans was applied. Automatic gain control (AGC) was set at 3E6 for full MS and 1E5 for MS/MS. The maximum IT was set at 20 ms for full MS and auto for MS/MS. The isolation window for MS/MS was set at 2.0 m/z.

### High performance liquid chromatography (HPLC)

A Waters e2695 HPLC and Photodiode array detector were used to assay the chromatographic peaks of WCWE. The chromatographic column was an Agilent ZORBAX SB-C18, 4.6×250mm, 5μm; the wave length was 270nm; the column temperature was 30℃; the flow velocity was 1.0 ml/min. The gradient elution was conducted with a mobile phase A (methyl alcohol) and a mobile phase B (1% glacial acetic acid).

### Statistical methods

All data was presented as a mean ± standard deviation (SD). A *Graphpad Prism 5.0* software (GraphPad Software, San Diego, CA) was used to analyze all data on the zebrafish. The *Ballgown* software was used to calculate the FPKM (Fragments Per Kilobase of gene/transcript model per Million mapped fragments) at the genetic and transcriptomic level. An independent-sample *t* test was used to compare the difference between the WCWE group and the negative control group. A *p* value less than 0.05 was considered to be statistically significant.

The resulting MS data were processed using Skyline software (v.3.6). Peptide settings: enzyme was set as Trypsin [KR/P], Max missed cleavage set as 2. The peptide length was set as 8-25, Variable modification was set as Carbamidomethyl on Cys and oxidation on Met, and max variable modifications were set as 3. Transition settings: precursor charges were set as 2, 3, ion charges were set as 1, 2, and ion types were set as b, y, p. The product ions were set from ion 3 to the last ion, and the ion match tolerance was set as 0.02 Da.

## Results

### Anti-angiogenic effects of WCWE

**Figure 1:**
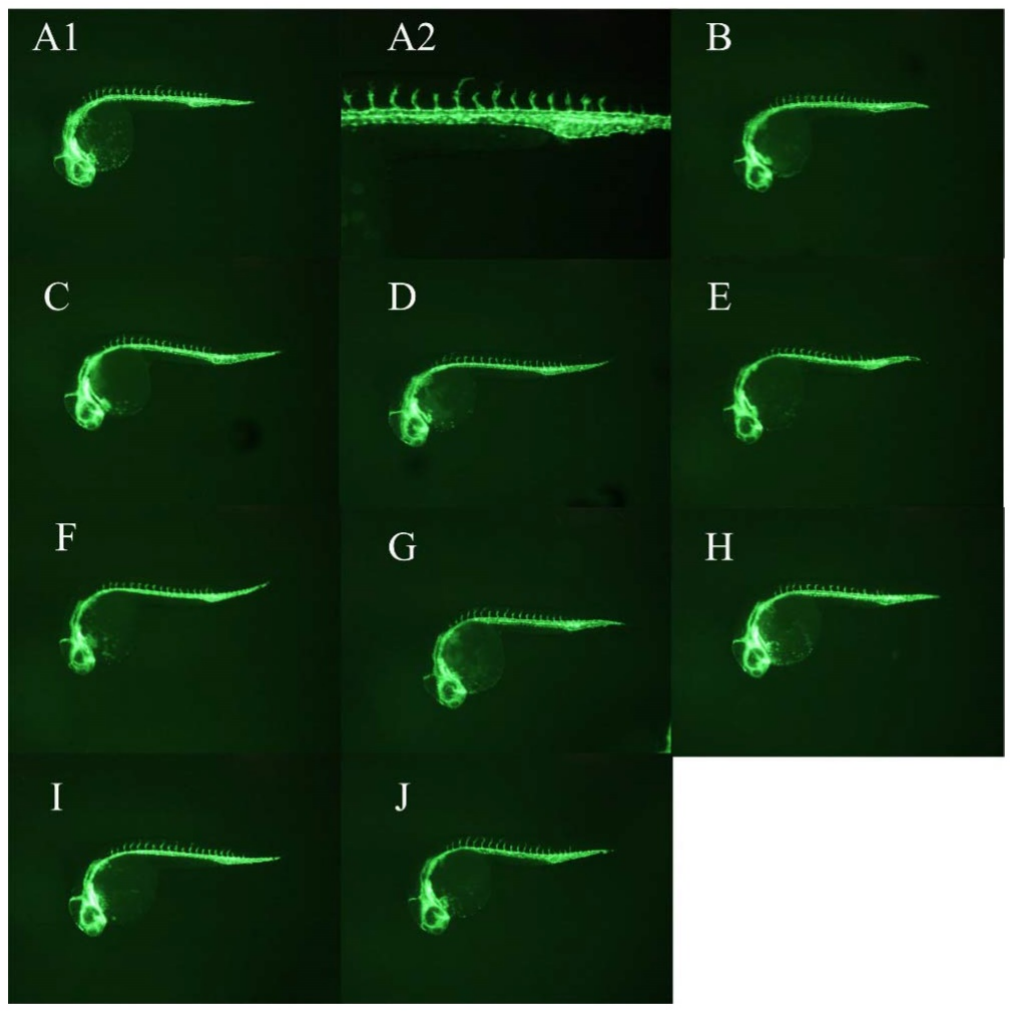
WCWE showed a significant anti-angiogenic effect in zebrafish. Compared with vehicle controls, the zebrafish embryo treated by WCWE showed a significant angiogenesis defect (panel A1, B-J). An image at higher magnification showed that the larvae treated with WCWE presents a lower number of complete ISVs (panel A2).

**Figure 2:**
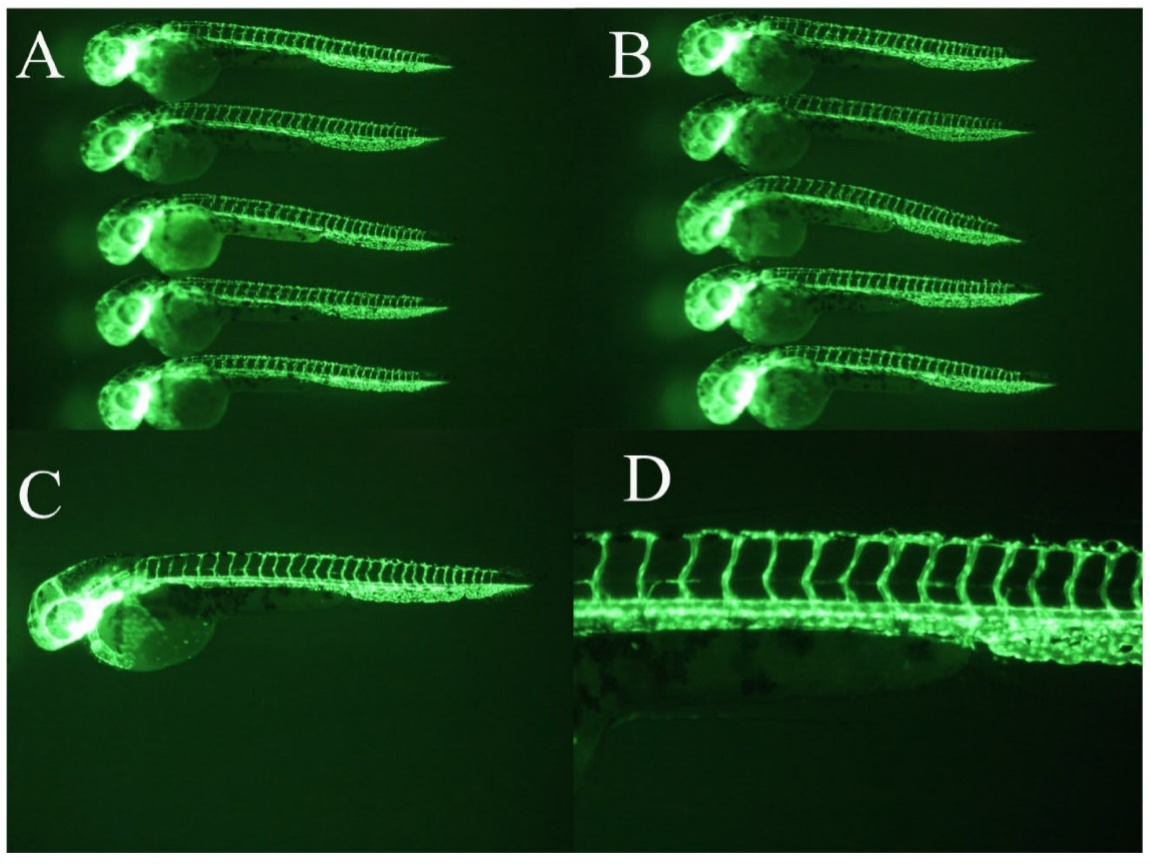
Vehicle controls. DMSO served as a vehicle control. Vehicle controls did not show a significant angiogenesis defect (panel A-D).

**Figure 3:**
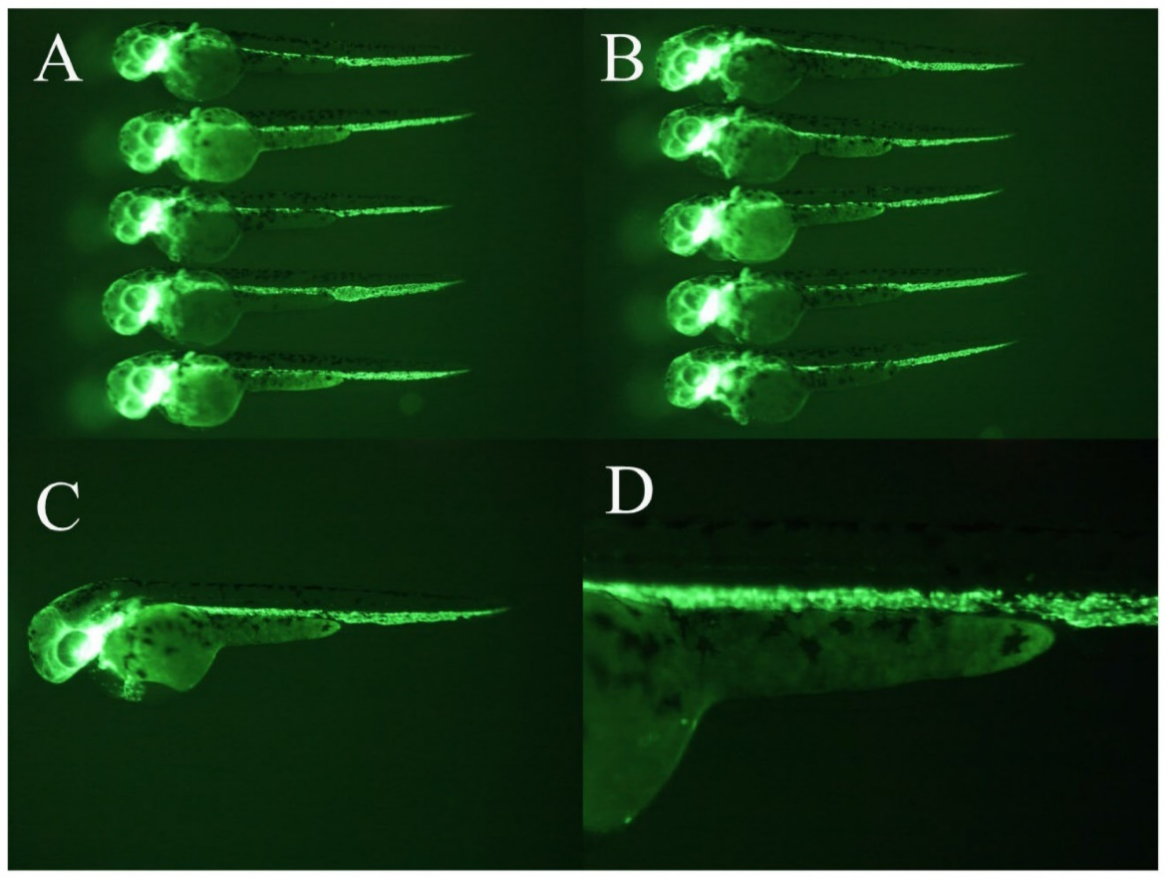
Positive controls. 5μg/ml PTK 787 showed a more potent anti-angiogenic effect than WCWE.

WCWE showed a significant anti-angiogenic effect in zebrafish (Figure 1-2), which was consistent with our previous results 5. 5μg/ml PTK 787 showed more potent anti-angiogenic effect than WCWE (Figure 3).

### mRNA-seq

**Figure 4:**
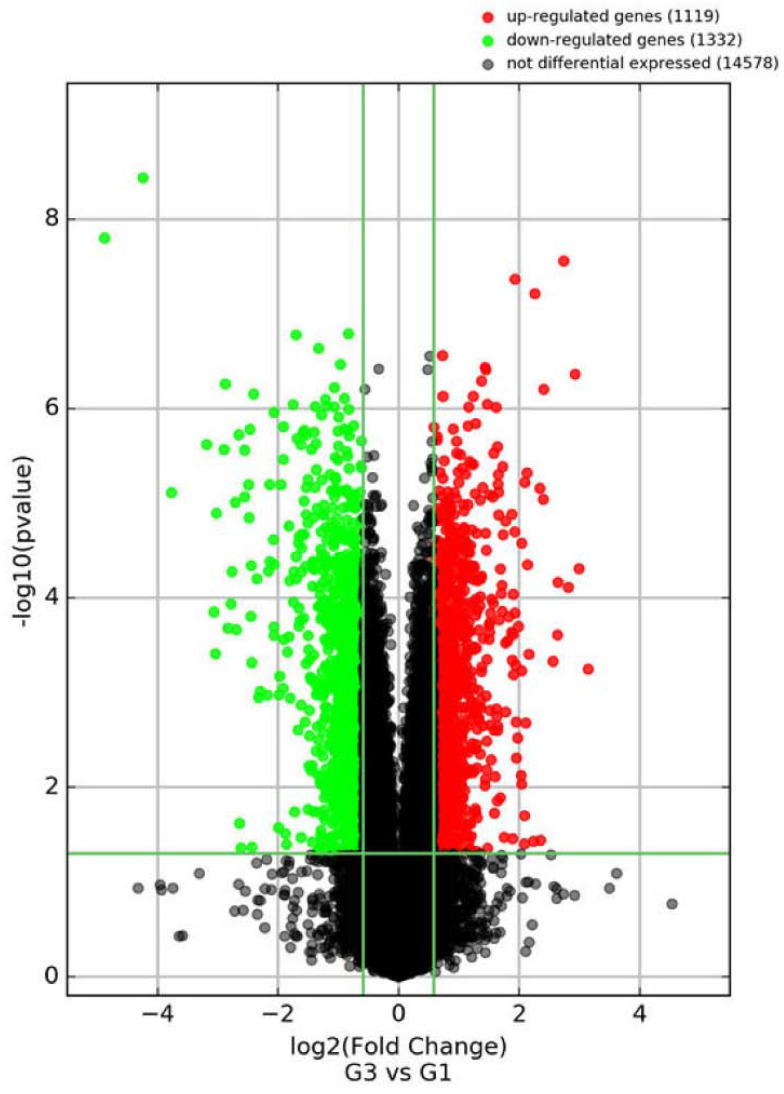
Volcano Plot. Volcano plot of the genes regulated by WCWE.

There were 1119 genes up-regulated and 1332 genes down-regulated by WCWE (Figure 4). The FPKM and the fold change of the ten significantly changed genes involved in proteasome signaling pathways are shown in Table 1.

### qRT-PCR assay

**Figure 5:**
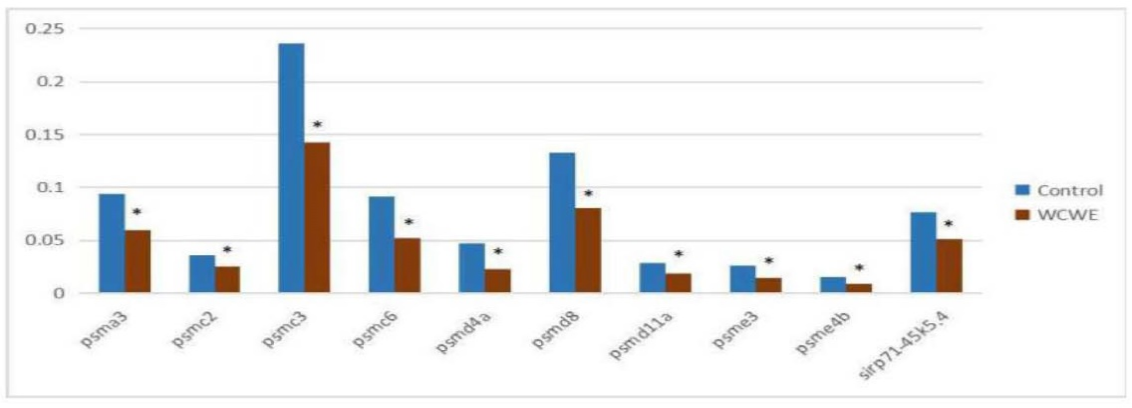
Results of qRT-PCR assay. The relative genetic expression levels of the ten genes involving proteasome signaling pathway. There was significant differences between the vehicle control group and the WCWE group in all ten genes (* indicates P < 0.05).

The results of qRT-PCR showed that the relative genetic expression levels of all ten genes involved in proteasome signaling were significantly down-regulated (Figure 5, Table S1, Figure S1-30), which was consistent with the results of the mRNA-seq assay.

### PRM assay

**Figure 6:**
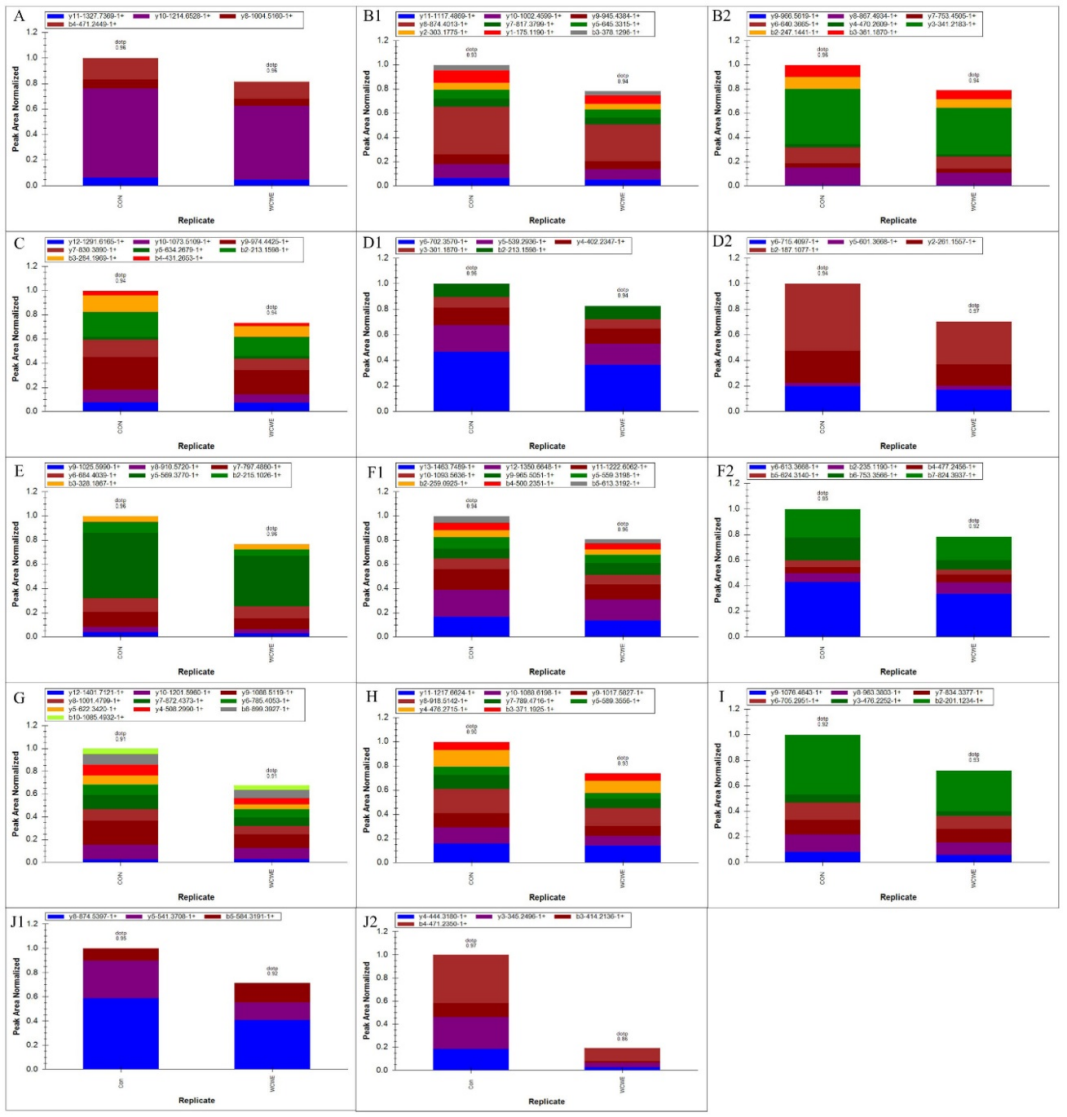
Results of PRM assay. Peptide fragments ion peak area distribution of the nine significantly changed proteins in the proteasome signaling and β-catenin (panel A: psmc6; panel B1-2: psmc2; panel C: psmd4a; panel D1-2: psmd7; panel E: psmc3; panel F1-2: psmc5; panel G: psmd1; panel H: psmd12; panel I: psmd6; panel J1-2: ctnnb2).

The results of the PRM assay showed that nine proteins involved in proteasome signaling were significantly down-regulated. The protein expression level of ctnnb2 was also significantly down-regulated (Figure 6).

### KEGG pathway analysis

**Figure 7:**
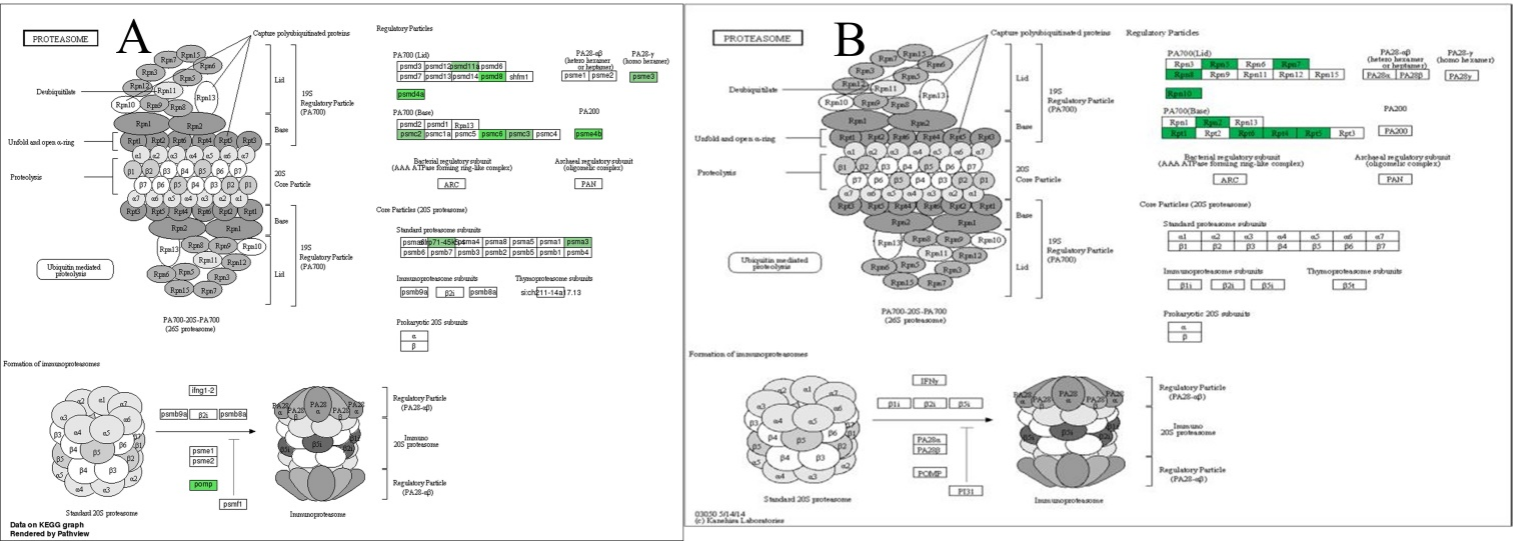
Proteasome signaling pathway regulated by WCWE. WCWE significantly down-regulated the genes involved in the proteasome signaling pathway. Green marked nodes are associated with down-regulated genes. Panel A is the KEGG pathway analysis based on the mRNA-seq assay. Panel B is the KEGG pathway analysis based on the PRM assay.

The KEGG pathway which was based on the results of mRNA-seq demonstrated that the significantly changed genes were involved in proteasome signaling (Figure 7, panel A). The KEGG pathway which was based on the results of the PRM assay demonstrated that the significantly changed proteins were involved in the regulatory particle of proteasome (Figure 7, panel B).

### HPLC

**Figure 8:**
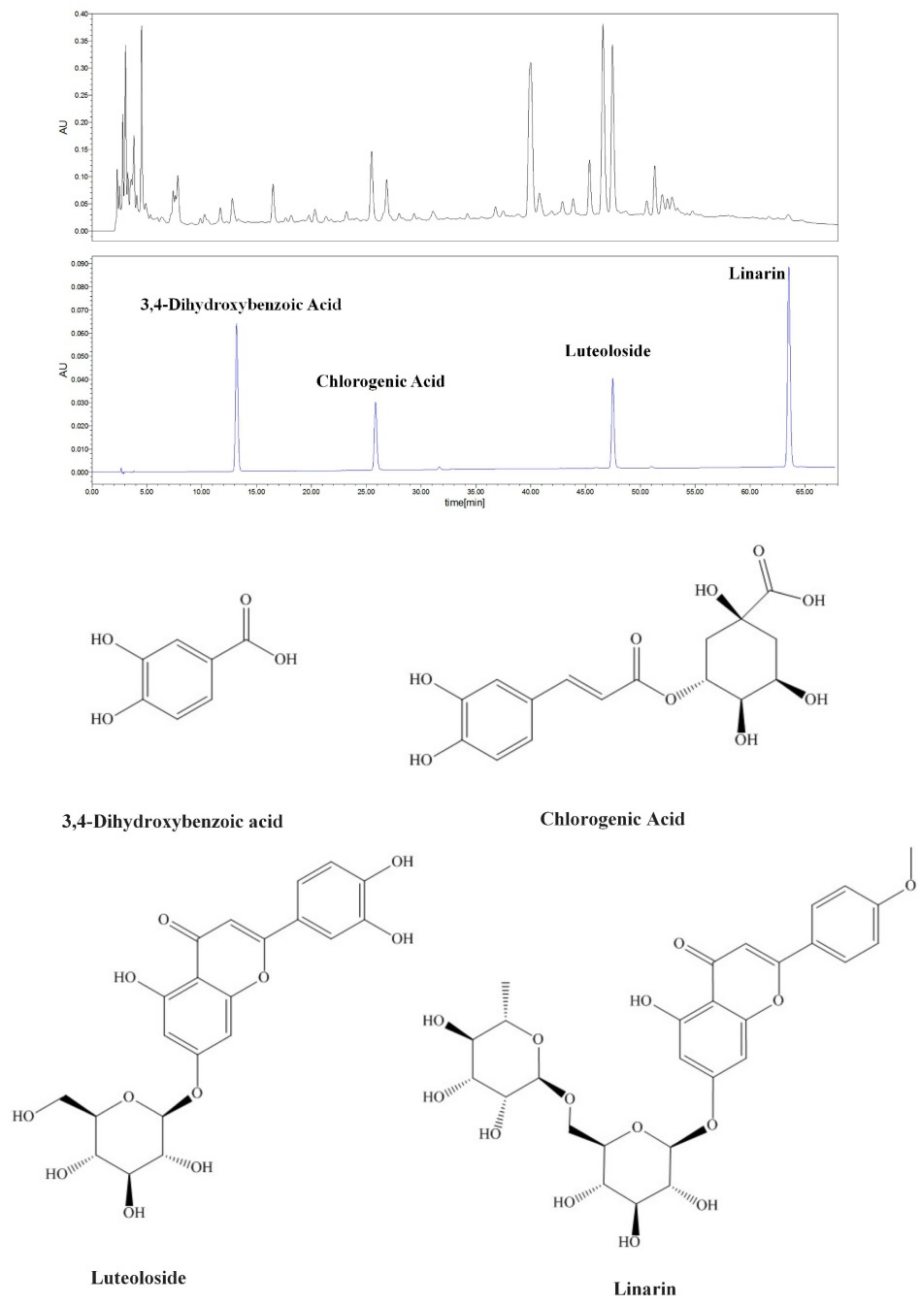
HPLC assay results. Four chromatographic peaks of WCWE were identified.

Four chromatographic peaks of WCWE were identified: 3,4-Dihydroxybenzoic acid (PubChem CDI: 72), Chlorogenic Acid (PubChem CID: 1794427), luteoloside (PubChem CID: 5280637), and linarin (PubChem CID: 5317025) (Figure 8).

## Discussion

After a half century of research, it is now widely accepted that angiogenesis is essential for the dissemination and establishment of tumor metastases 6. In the early 1990s, Weidner et al., 7 positively demonstrated that the amount of neovascularization measured directly in histologic sections of breast carcinoma correlates with the presence of metastasis. The expression levels of classic angiogenic effectors such as VEGF have been reviewed in our previous research 5. It is the first time that the zebrafish model offers researchers the opportunity to gain knowledge on how *wild chrysanthemum* inhibits angiogenesis by regulating proteasome signaling* in vivo*, which provides new translational insights into the therapeutic potential of *wild chrysanthemum* to help cancer patients.

Proteasome, also known as 26S proteasome complex, consists of a lid, a base, and the core. It engages in an ATP-dependent proteolytic degradation of a variety of oncoproteins, transcription factors, cell cycle specific cyclins, cyclin-dependent kinase inhibitors, ornithine decarboxylase, and other key regulatory cellular proteins. Therefore, it regulates either directly or indirectly many important cellular processes 8. The involvement of proteasome in the degradation of cancer-related proteins has been extensively studied 9. Many researchers believe in that targeting proteasome is a feasible and valuable approach in the treatment of cancer. The success of the first-in-class proteasome inhibitor, such as bortezomib and carfilzomib - is inspiring scientists to explore other targets in the proteasome signaling pathway 10,11. We have known that the expression levels and activity of many proteins are controlled through regulated ubiquitination and subsequent destruction by the 26S proteasome. As a major protein quality and quantity control system, the ubiquitin proteasome system (UPS) has a significant impact on angiogenic regulator proteins.

It is notable that proteins of the proteasome regulatory particle were significantly down-regulated by WCWE, such as PSDM7. PSMD7 is reported to be overexpressed in most carcinoma cells and its down-regulation contributed to decelerated tumor growth, inhibition of proteasomal function, induced cell apoptosis and attenuated activity of the mTOR/p70S6K pathway 12. The reduced expression level of the proteasome regulatory particle means that proteasome activities were inhibited. Proteasome inhibitors have been used in the clinic to treat different types of cancer 13,14. Many proteasome inhibitors, such as bortezomib 15 and lactacystin 16, have been proven to be associated with anti-angiogenesis. It is also notable that β-catenin were significantly down-regulated by WCWE at the protein level. β-catenin is a pivotal component of the Wnt signaling pathway; and it is regulated at three hierarchical levels: protein stability, sub-cellular localization, and transcriptional activity 17. In the tissues of patients with non-small cells lung cancer, the increase in β-catenin content is associated with an increase in the number of proteasomes 17. It has been reported that the detergent-insoluble nuclear component of β-catenin displays glycogen synthase kinase 3β (GSK-3β) and APC-independent proteasome sensitivity 18. Although regulation of β-catenin proteasome sensitivity and the contribution of this process to β-catenin function may be very complex, our research results showed that WCWE may significantly inhibit β-catenin activity by the role similar to proteasome inhibitors.

We cannot technically say the present article is novel as we have first reported the phenotype that WCWE has an anti-angiogenic effect in our previous article 5. But the present article greatly improves our understanding of the anti-cancer potential of *wild chrysanthemum. Wild chrysanthemum* has been considered to be a safe TCM in respected TCM books, and we did not notice any toxic effect in zebrafish given WCWE. Consequently*,* we think the anti-angiogenic effect was not a result from a toxic effect of *Wild chrysanthemum. Wild chrysanthemum* contains essential oils 0.60-1.29% (camphor, α-pinene, carvone, eucalyptol, borneol), and flavonoids (acaciin, linarin), chrysanthemin 0.42-0.45%, luteolin, and acacetin 19. We tried to identify more of the ingredient compounds of *wild chrysanthemum* as we could; and therefore, we used reference samples different from our previous study 5. This results in inconsistent HPLC assay findings. The ingredient compounds identified from WCWE have not been fully investigated and we are now continuing to carry out more experiments to determine which ingredient compound contributes to the effects of WCWE.

In ancient TCM theory, *wild chrysanthemum* has “*heat-clearing and detoxicating*” therapeutic action and enters the channels of the liver and the lung. Based on this ancient meridian entry theory, Chinese researchers noticed that it could inhibit the growth of human hepatocellular carcinoma MHCC97H cells 20 and thereafter, more research explored the anti-cancer potency of *wild chrysanthemum.* It is reported that *wild chrysanthemum* flavonoids could significantly inhibit the proliferation and induce the apoptosis of human lung cancer A549 cells and human osteosarcoma Saos-2 cells 21,22. Researchers have screened the anti-cancer active fractions of *wild chrysanthemum in vitro* and their conclusion was “the antitumor activity sites were the petroleum ether and ethyl acetate fractions” 23. *Wild chrysanthemum* is traditionally decocted together with other TCM with water and this is the reason why we used water extract. As *wild chrysanthemum* contains a large number of ingredient compounds, it is unclear which specific ingredient compound has potent anti-cancer properties. In the opinions of the authors of the present article, confirmation of the anti-cancer properties of *wild chrysanthemum* is currently much more important than determination of the exact working compounds. Therefore, this study used WCWE rather than the ingredients of *wild chrysanthemum* to conduct experiments *in vivo*. Apparently, there is a lot of work to do before we can determine the exact anti-angiogenic ingredient compounds of *wild chrysanthemum*.

## Conclusions

*Wild chrysanthemum* has a significant anti-angiogenic effect in zebrafish and it has an inhibitory action on the regulatory particle of the proteasome. The mechanisms underlying the anti-angiogenic effects of *wild chrysanthemum* may be related to the down-regulation of proteasome/β-catenin signaling in zebrafish.

## Supplementary Material

Supplementary figures and table.

## Figures and Tables

**Table 1:** The FPKM and the fold change of the significantly changed genes involved in proteasome signaling pathway

Gene name	FPKM of the WCWE group	FPKM of the vehicle control group	fold change	*P* value
psme3	3.627	4.406	0.583	2.14E-05
psmc6	4.986	5.841	0.553	0.0005
psmc3	5.453	6.124	0.628	0.0050
psmc2	5.400	6.036	0.644	0.0132
si:rp71-45k5.4	5.746	6.359	0.654	4.256E-06
psmd4a	3.839	4.922	0.472	0.0056
psmd8	4.930	5.868	0.522	0.0002
psmd11a	3.421	4.138	0.609	7.963E-05
psme4b	2.421	3.504	0.472	0.003
psma3	5.826	6.624	0.575	0.0040

## Abbreviations

DMSO: dimethyl sulfoxide
FPKM: Fragments Per Kilobase of gene/transcript model per Million mapped fragments
hpf: h post-fertilization
HPLC: high performance liquid chromatography
PRM: parallel reaction monitor
qRT-PCR: quantitative real-time PCR
SD: standard deviation
TCM: Traditional Chinese Medicine
UPS: ubiquitin proteasome system
WCWE: *Wild chrysanthemum* water extract
