# Supplementary material for: Screening Study on the Anti-Angiogenic Effects of Traditional Chinese Medicine - Part II: Wild Chrysanthemum
Source: J Cancer. 2021 Jan 1;12(1):124–33. doi: 10.7150/jca.52971 (PMC7738828; doi:10.7150/jca.52971)
Supplement: Supplementary file 1 — Supplementary figures and table. [file jcav12p0124s1.pdf]

Table S1 Results of qRT-PCR assay

Gene name	Relative expression of the Control group	Relative expression of the WCWE group	P value
psme3	0.0262±0.00127	0.0144±0.00151	0.00049
psmc6	0.0911±0.00300	0.0524±0.00272	0.00008
psmc3	0.2363±0.00874	0.1427±0.00961	0.00023
psmc2	0.0356±0.00107	0.0257±0.00242	0.00295
si:rp71-45k5.4	0.0768±0.00358	0.0515±0.00166	0.00038
psmd4a	0.0476±0.00157	0.0229±0.00101	0.00002
psmd8	0.1327±0.00833	0.0811±0.00494	0.00086
psmd11a	0.0284±0.00106	0.0190±0.00035	0.00012
psme4b	0.0156±0.00095	0.0092±0.00016	0.00028
psma3	0.0934±0.00223	0.0592±0.00234	0.00005

Figure S1 Amplification Plot psma3

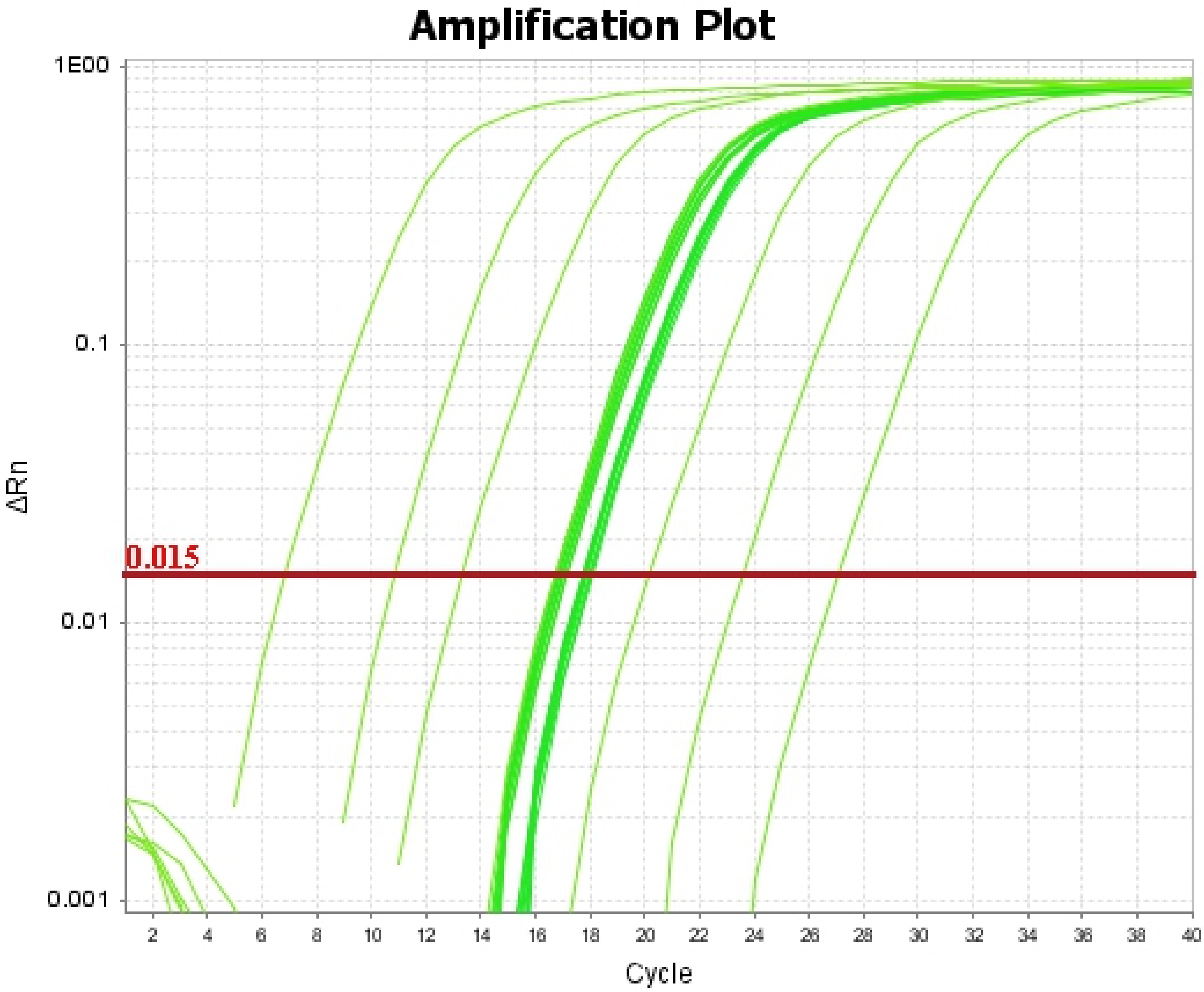

Figure S2 Amplification Plot psmc2

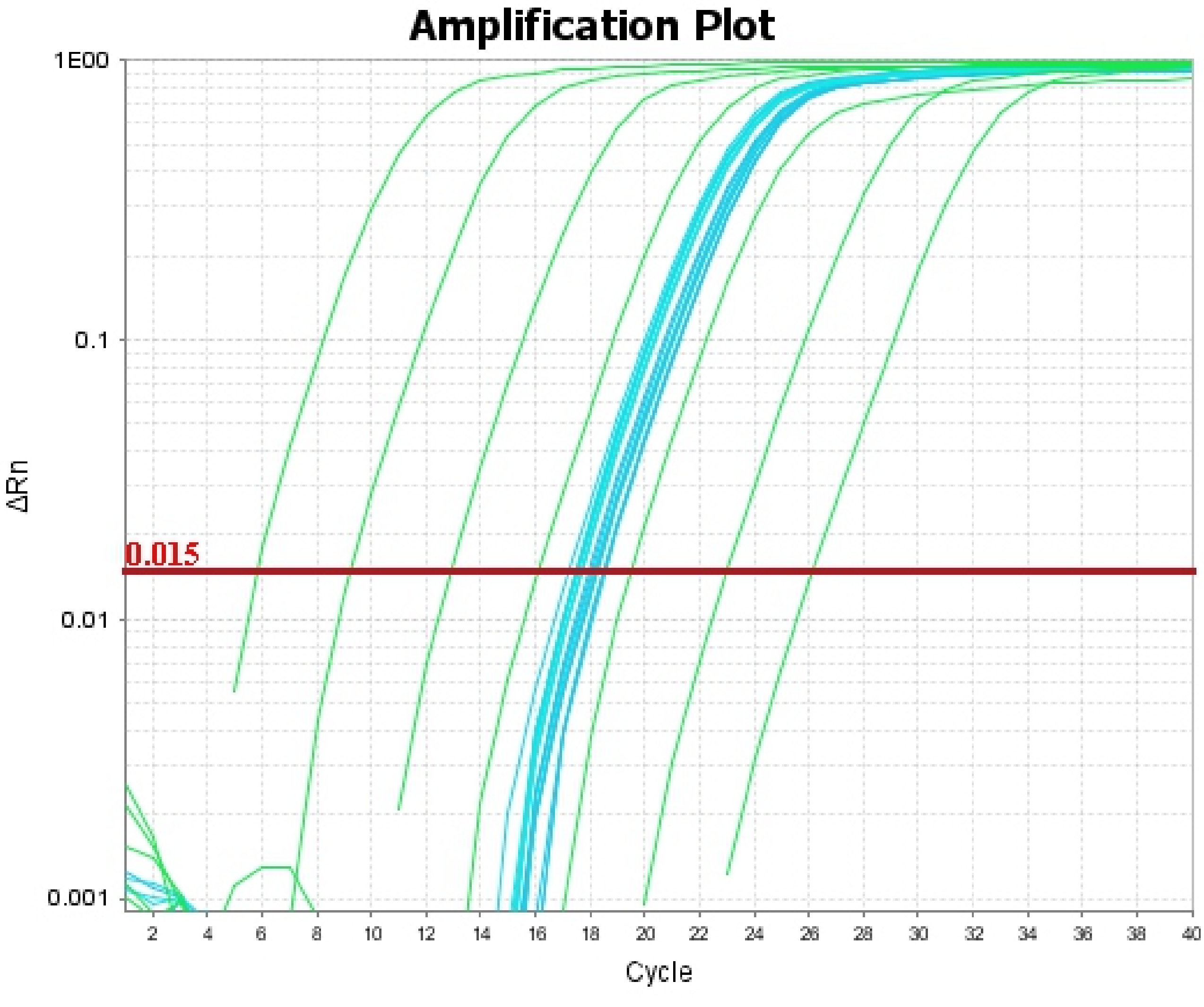

Figure S3 Amplification Plot psmc3

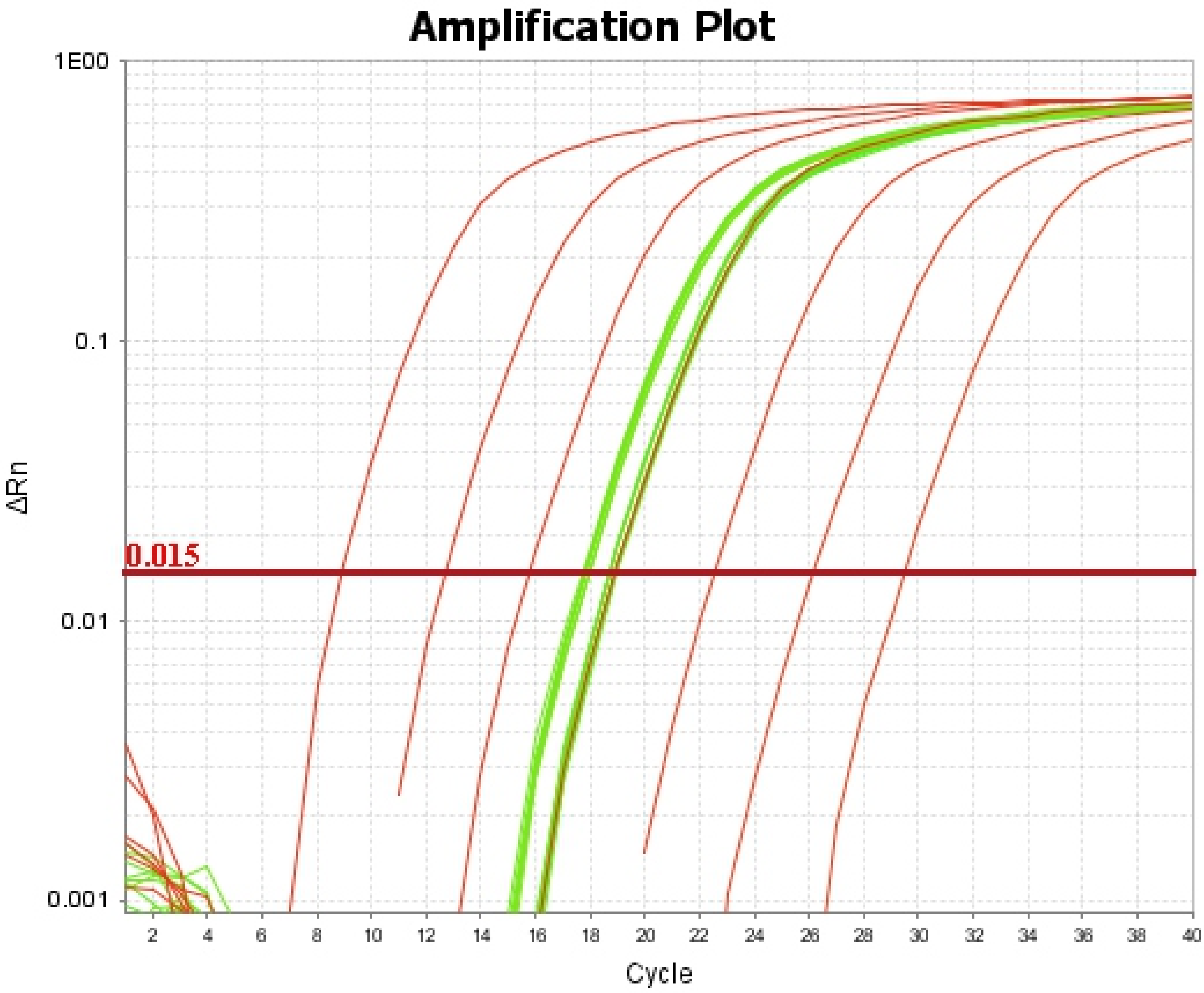

Figure S4 Amplification Plot psmc6

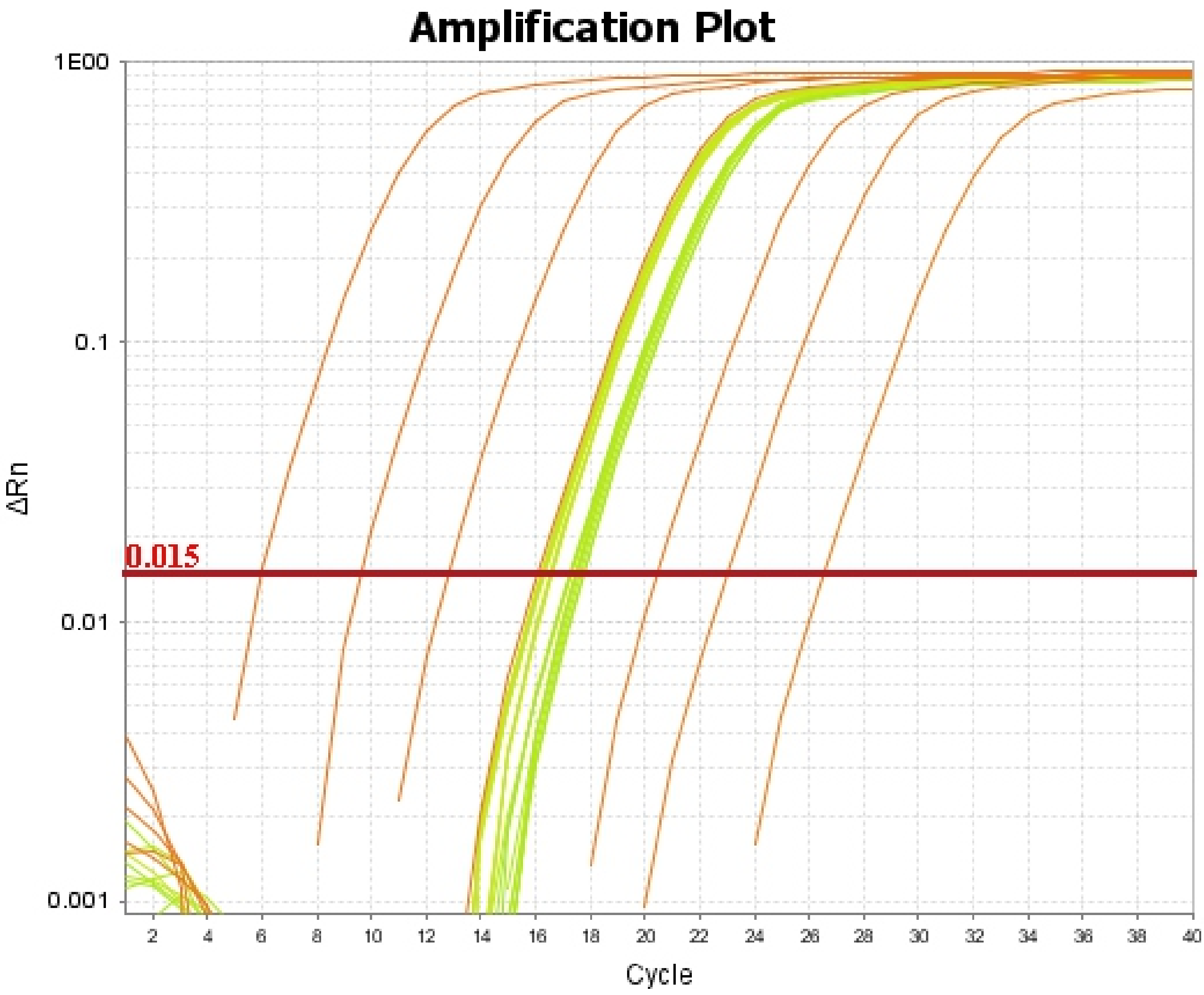

Figure S5 Amplification Plot psmd4a

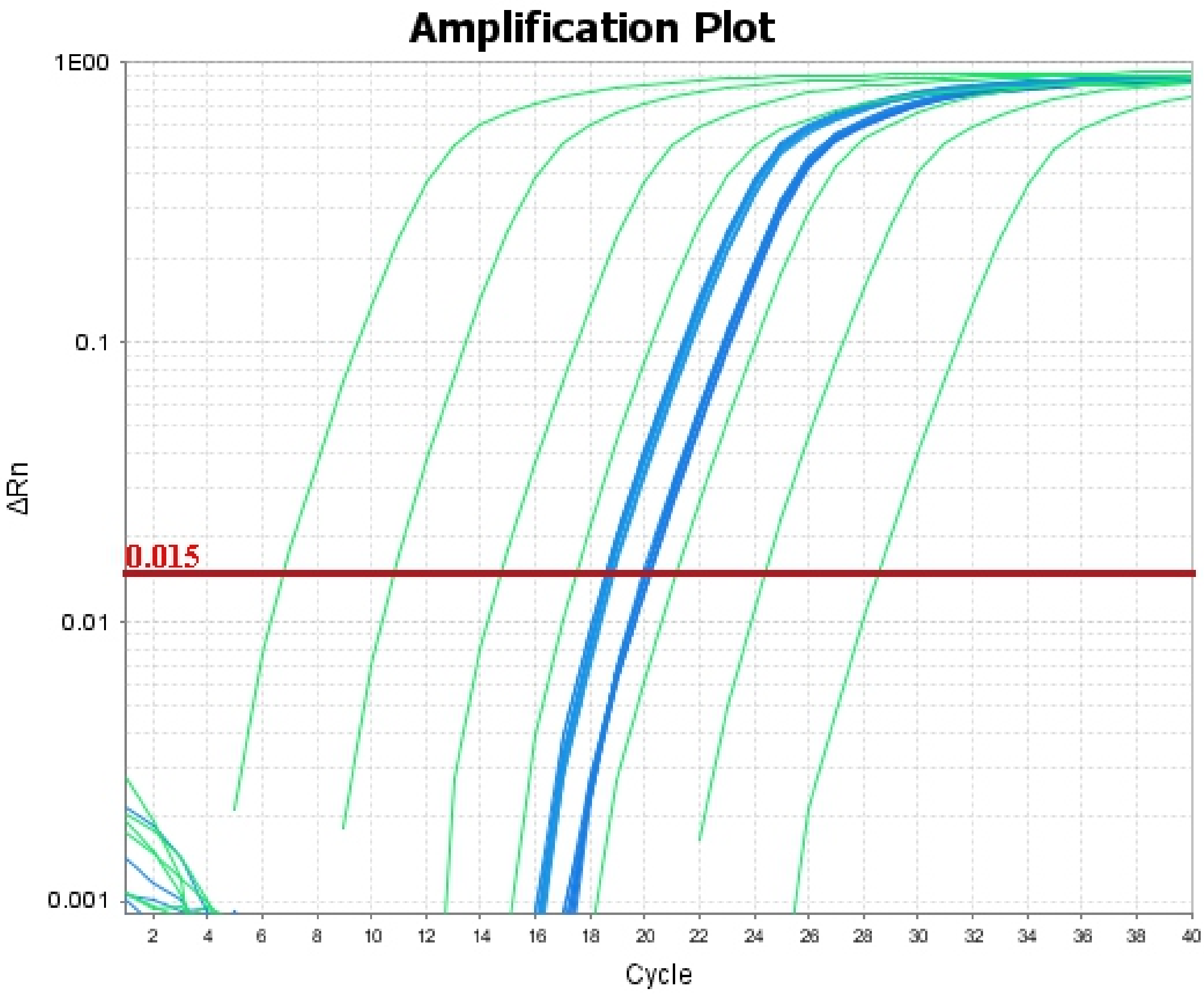

Figure S6 Amplification Plot psmd8

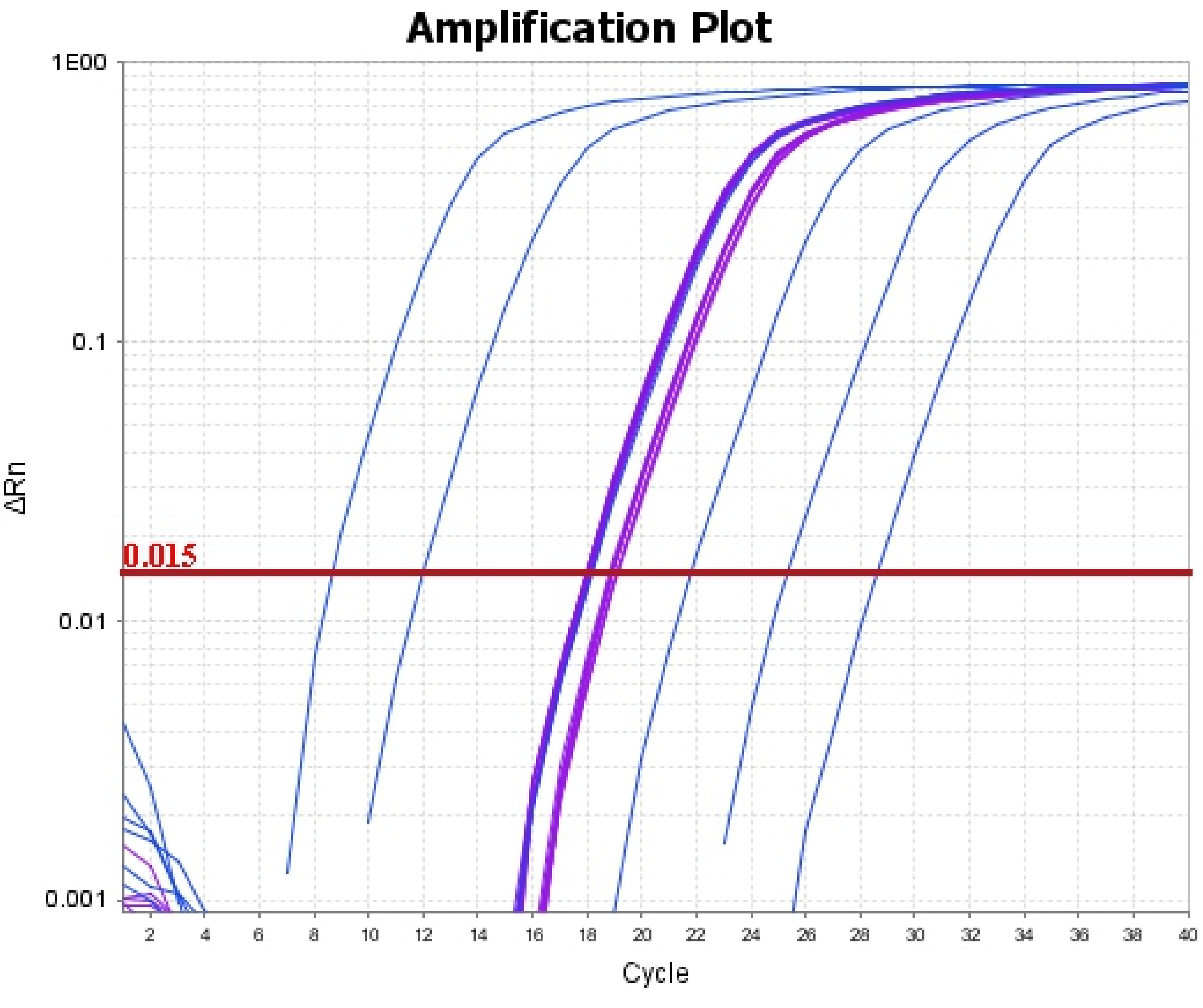

Figure S7 Amplification Plot psmd11a

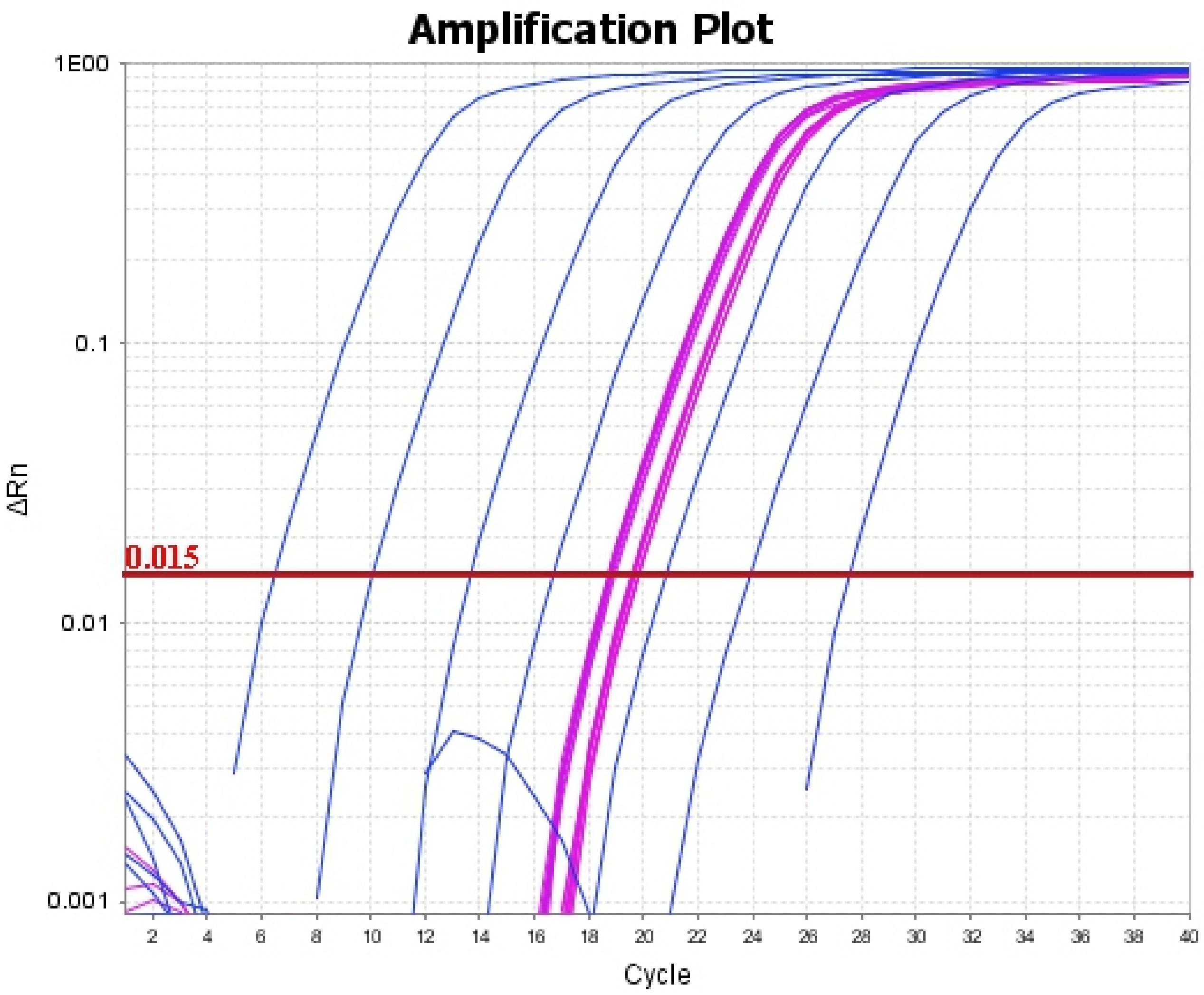

Figure S8 Amplification Plot psme3

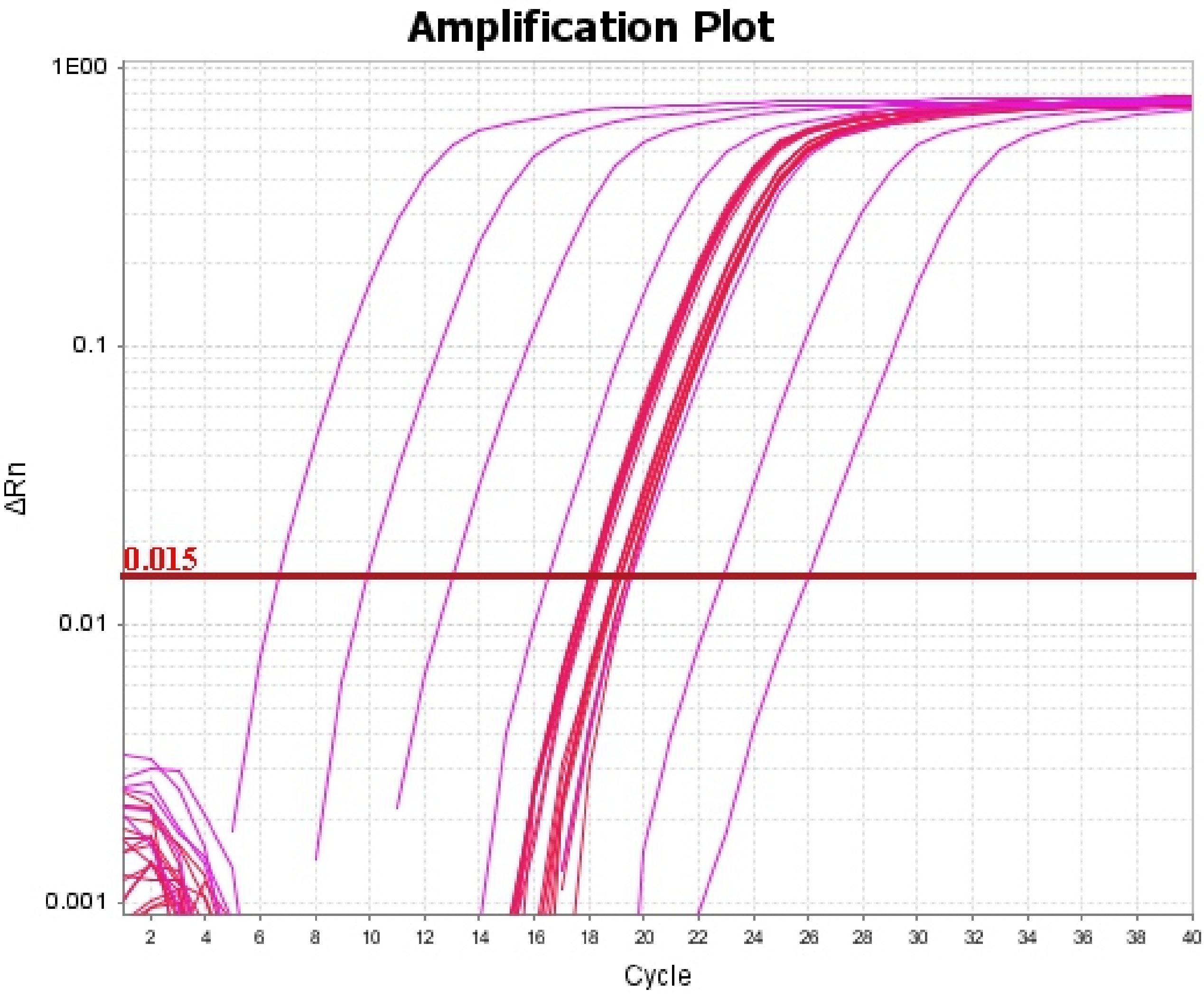

Figure S9 Amplification Plot psme4b

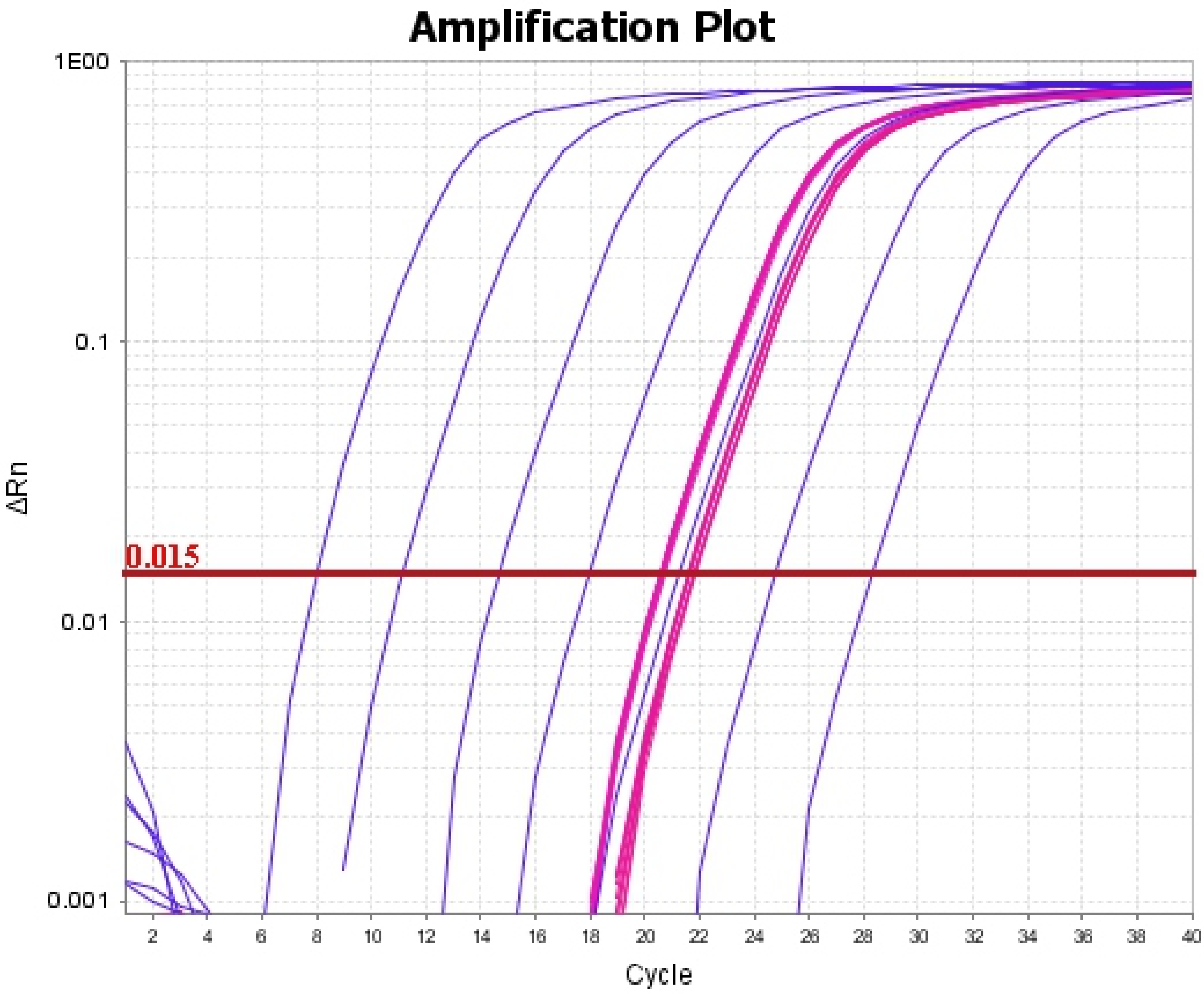

Figure S10 Amplification Plot sirp71-45k5.4

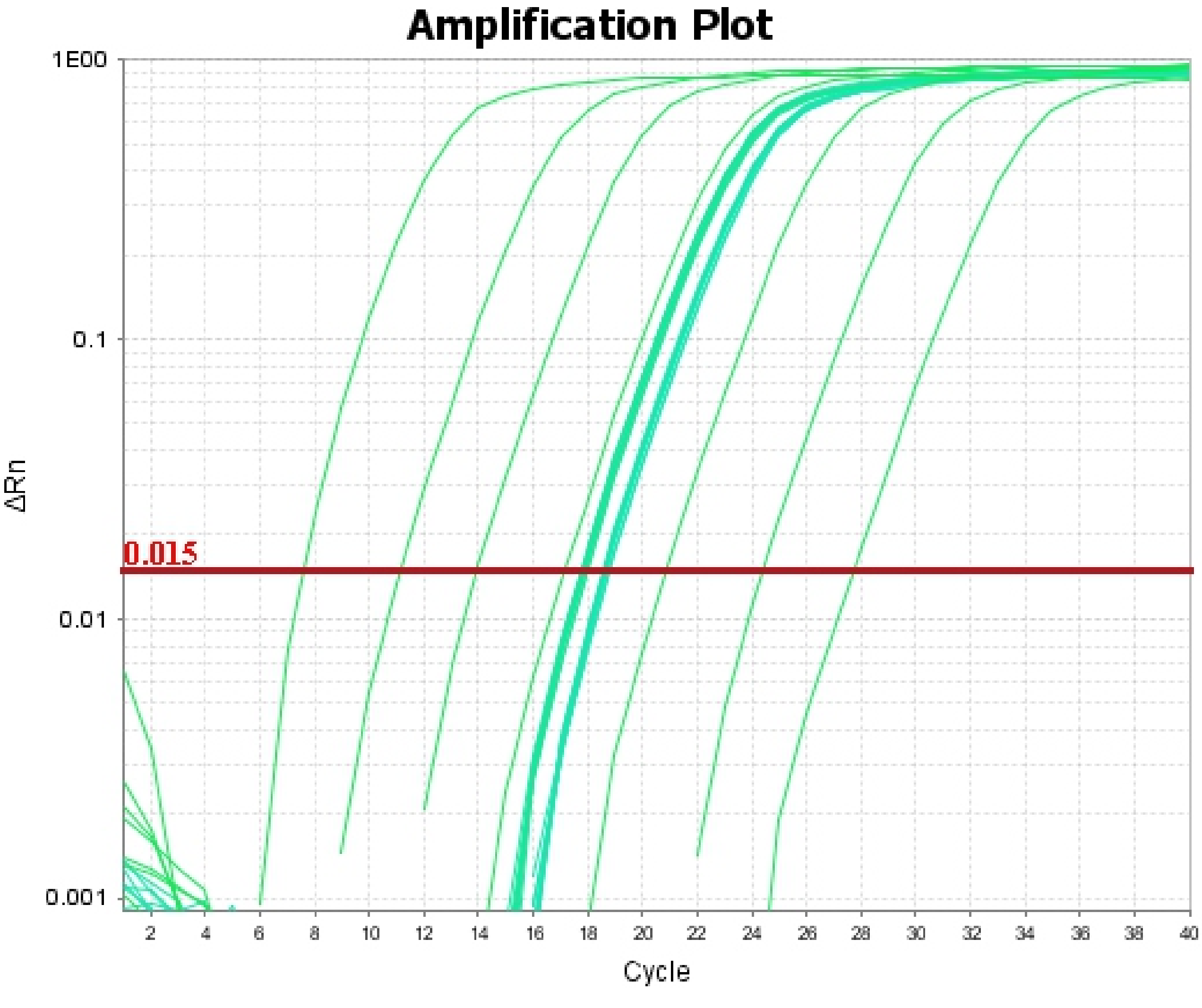

Figure S11 Melt Curve Plot psma3

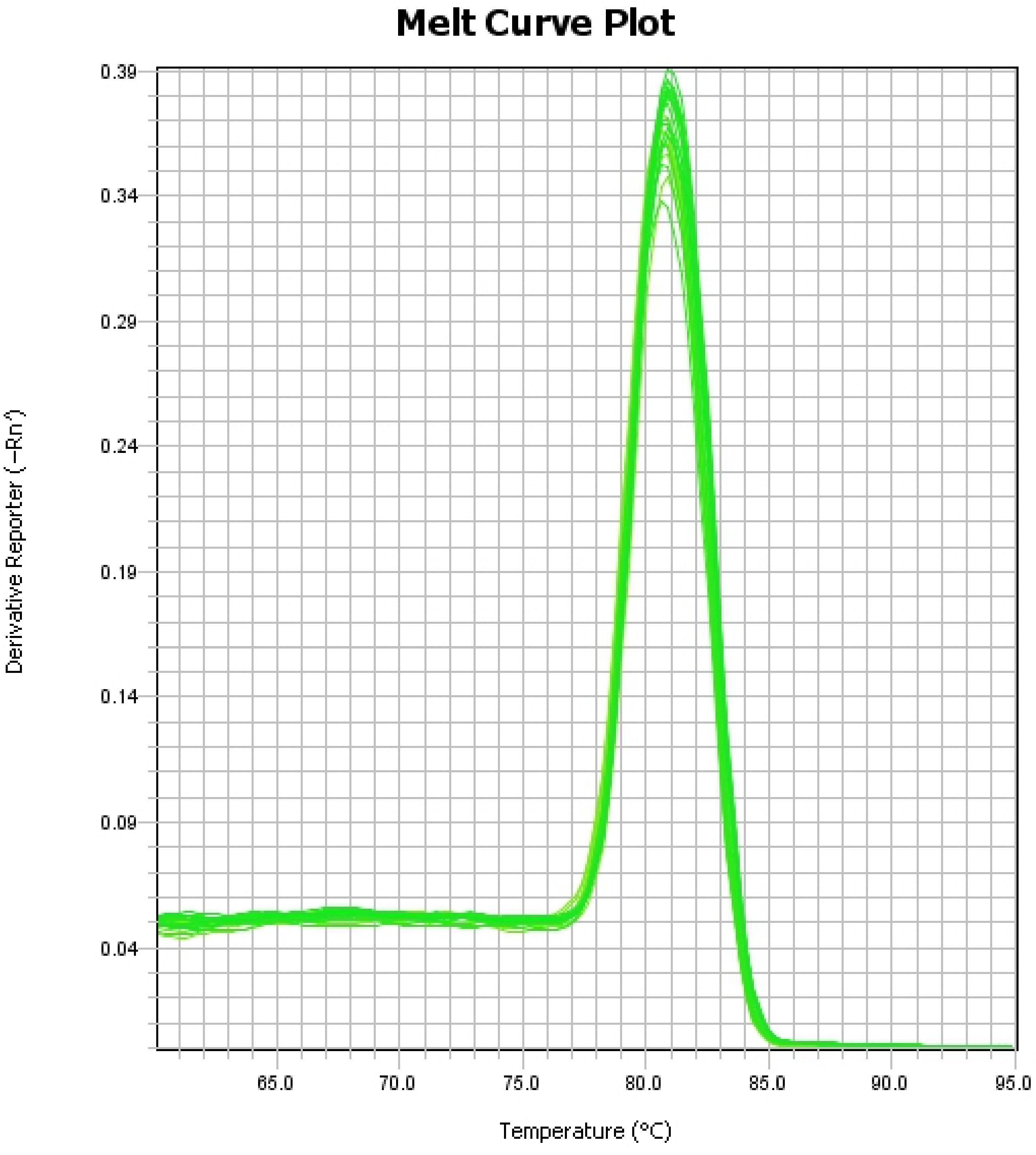

Figure S12 Melt Curve Plot psmc2

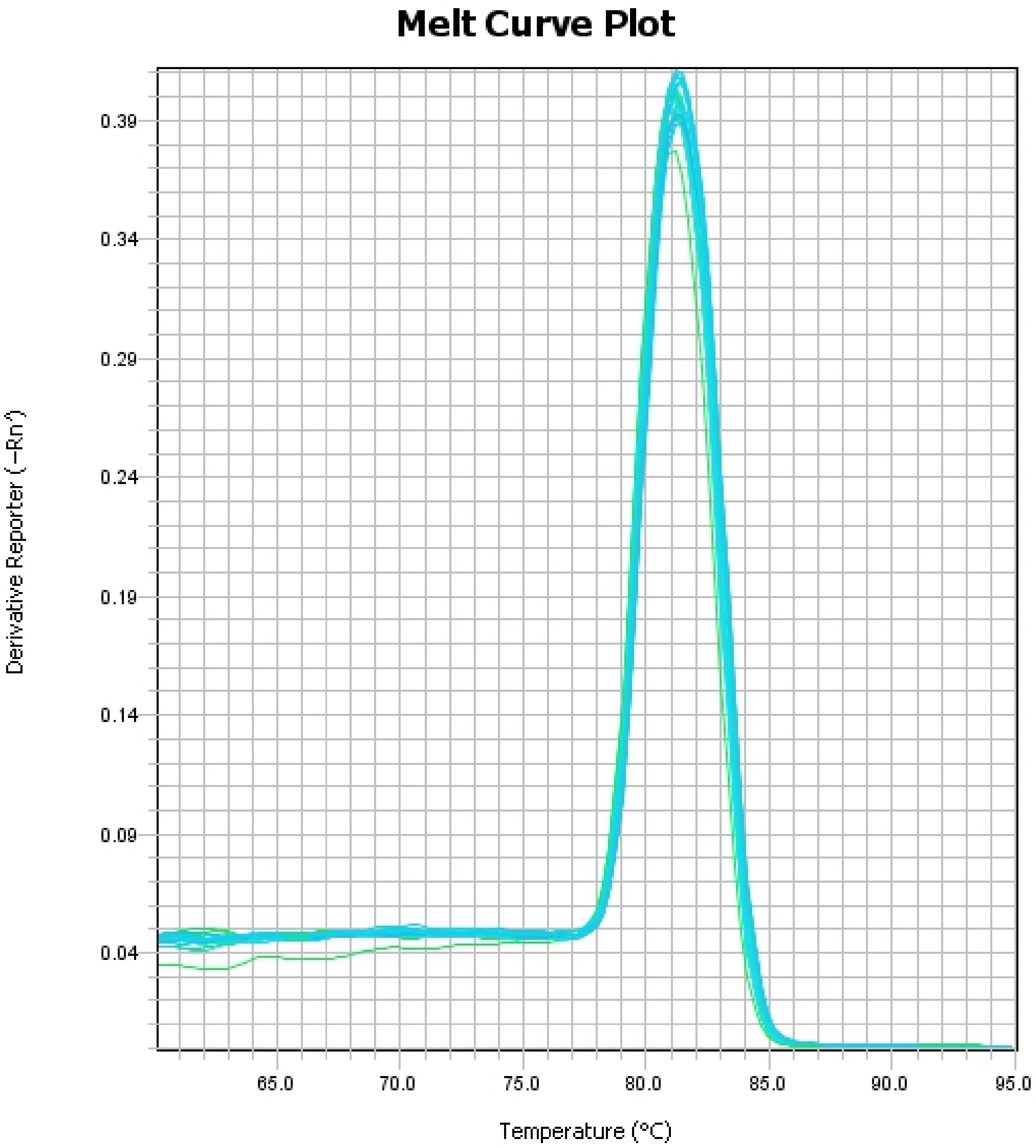

Figure S13 Melt Curve Plot psmc3

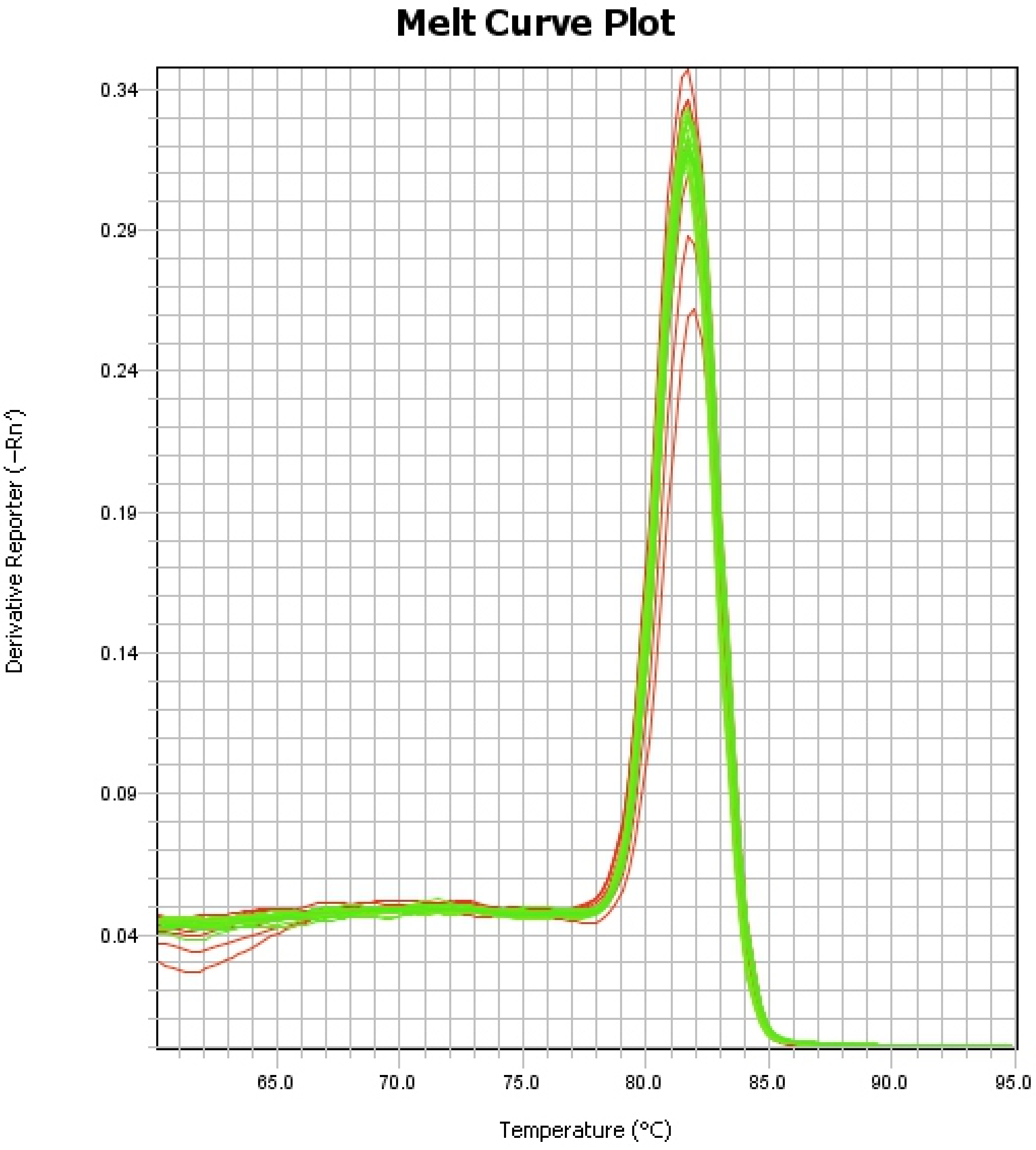

Figure S14 Melt Curve Plot psmc6

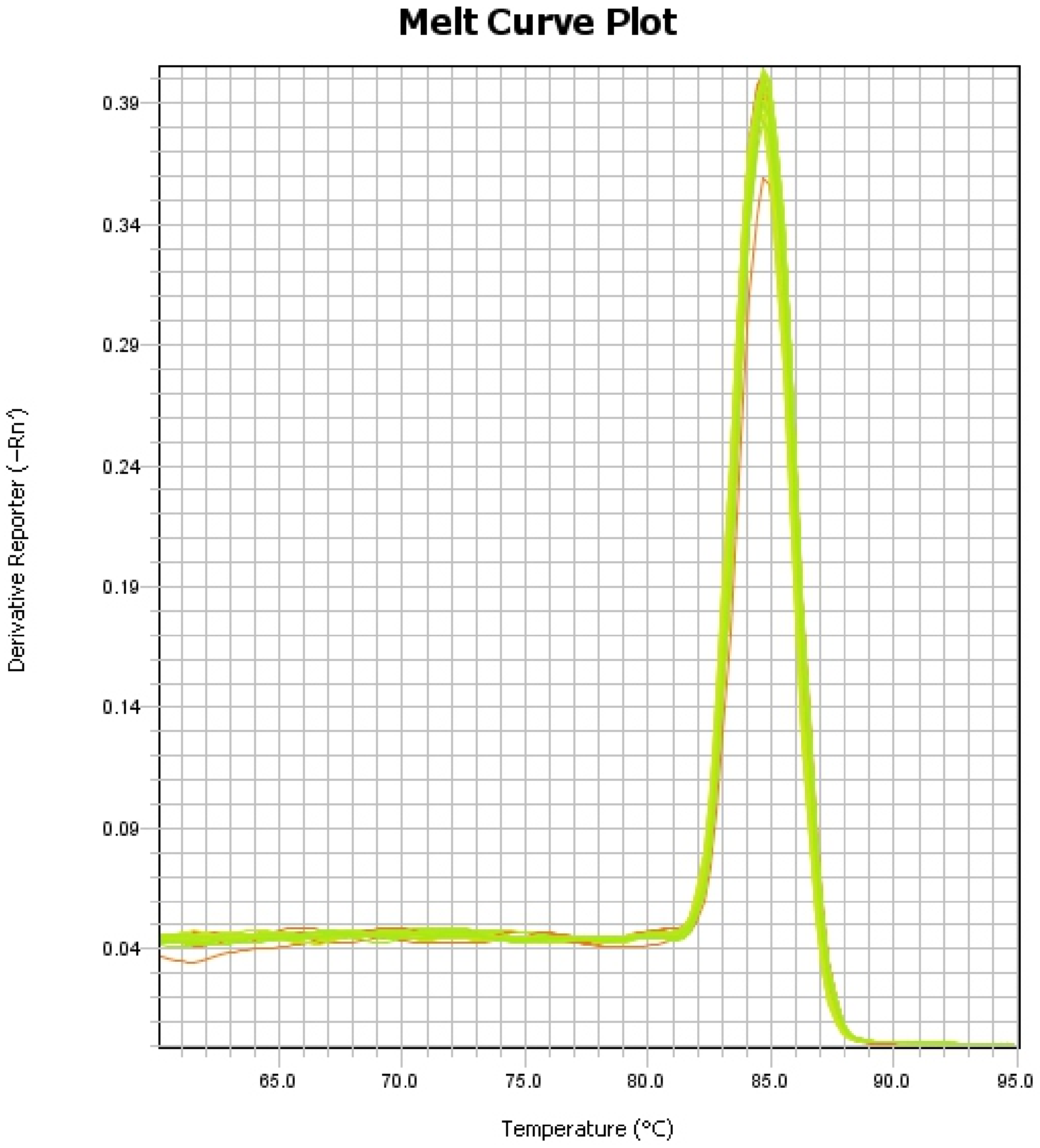

Figure S15 Melt Curve Plot psmd4a

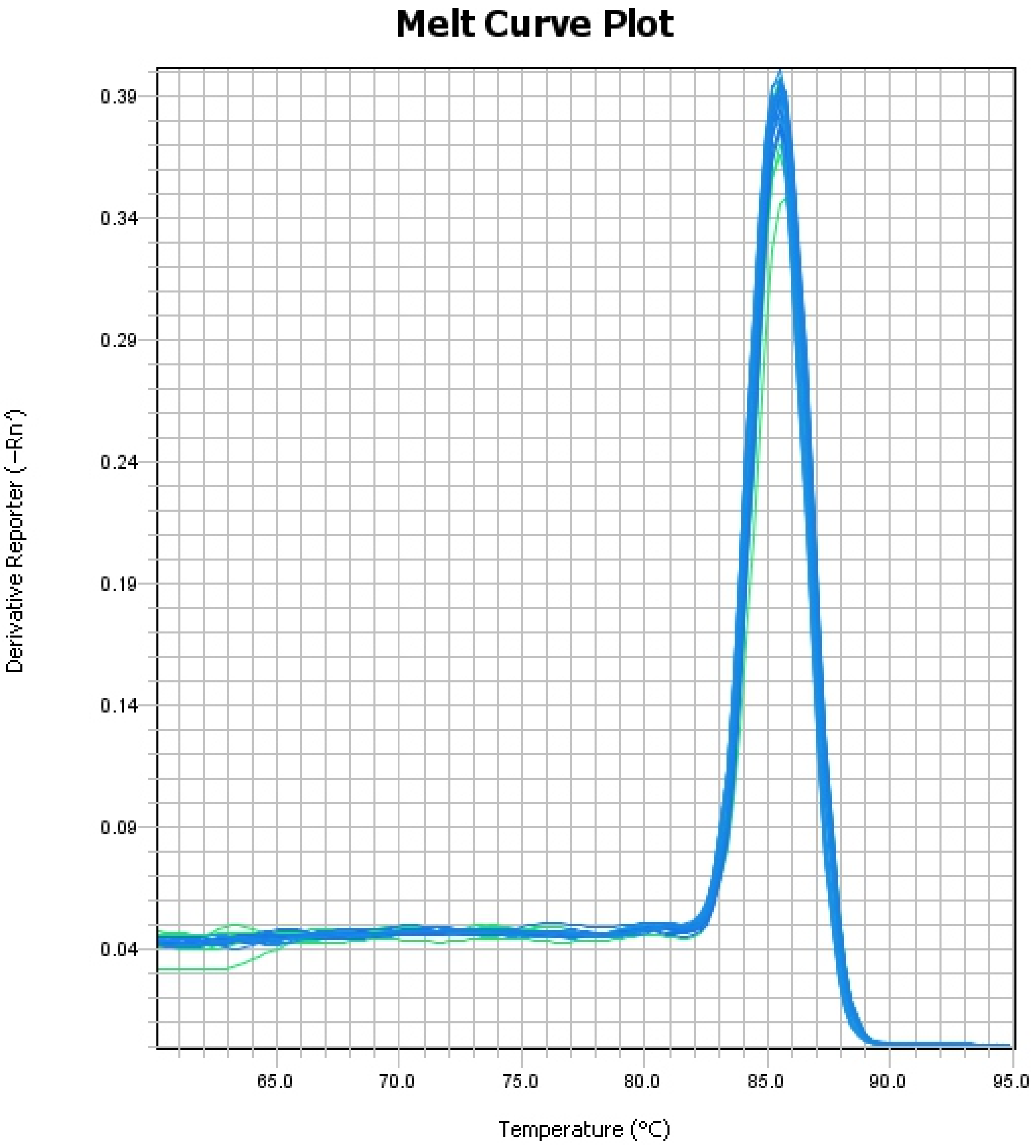

Figure S16 Melt Curve Plot psmd8

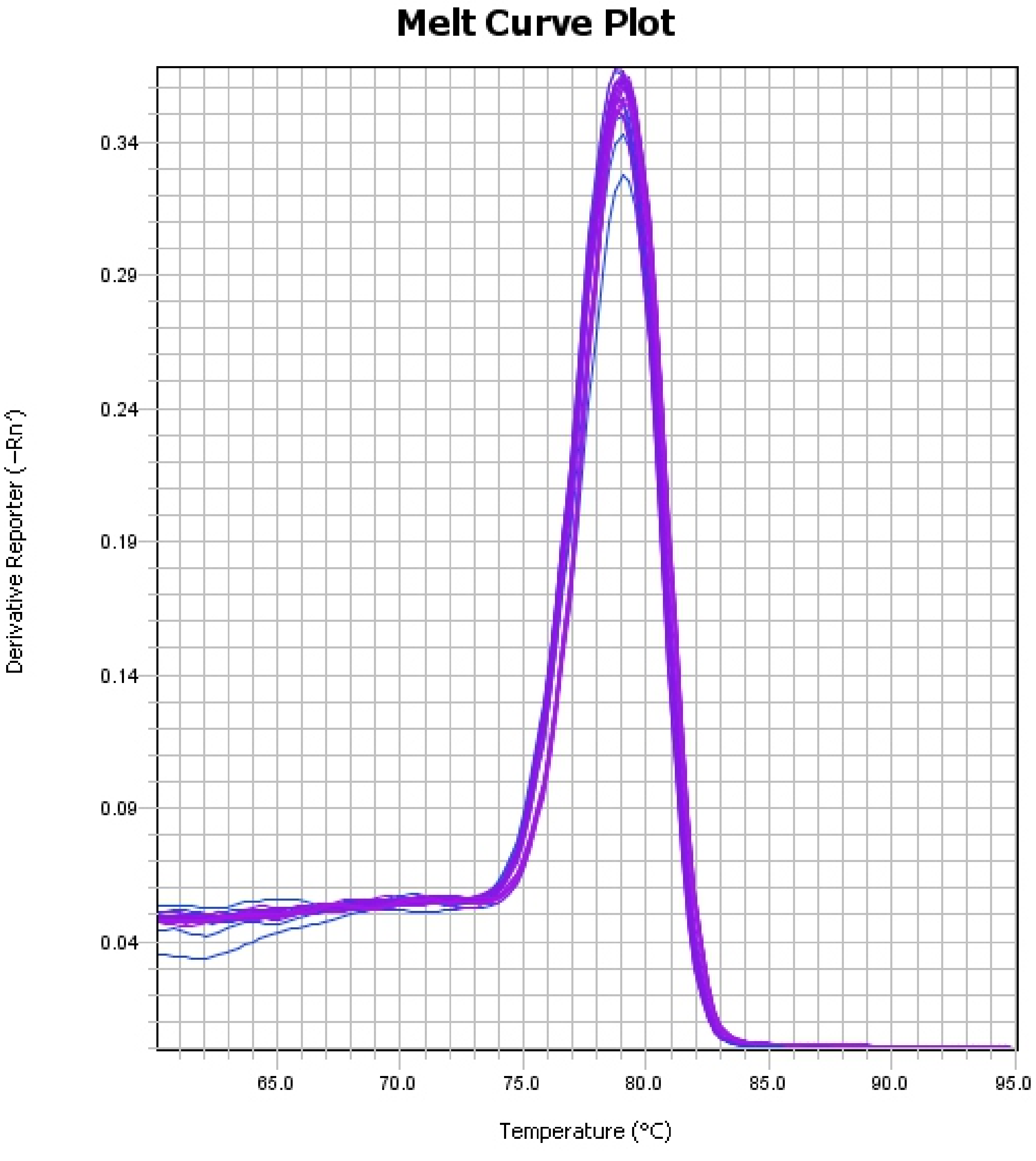

Figure S17 Melt Curve Plot psmd11a

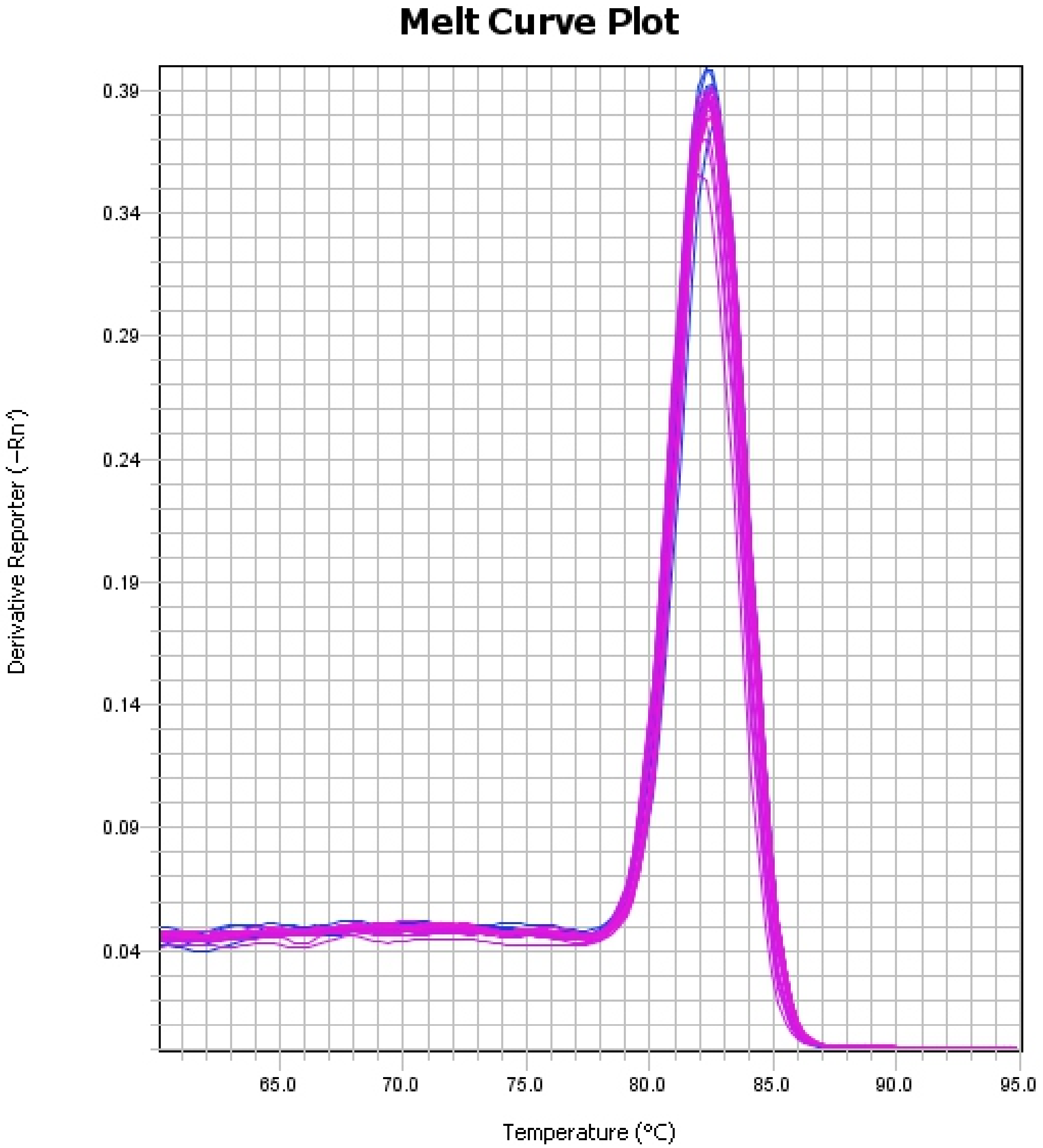

Figure S18 Melt Curve Plot psme3

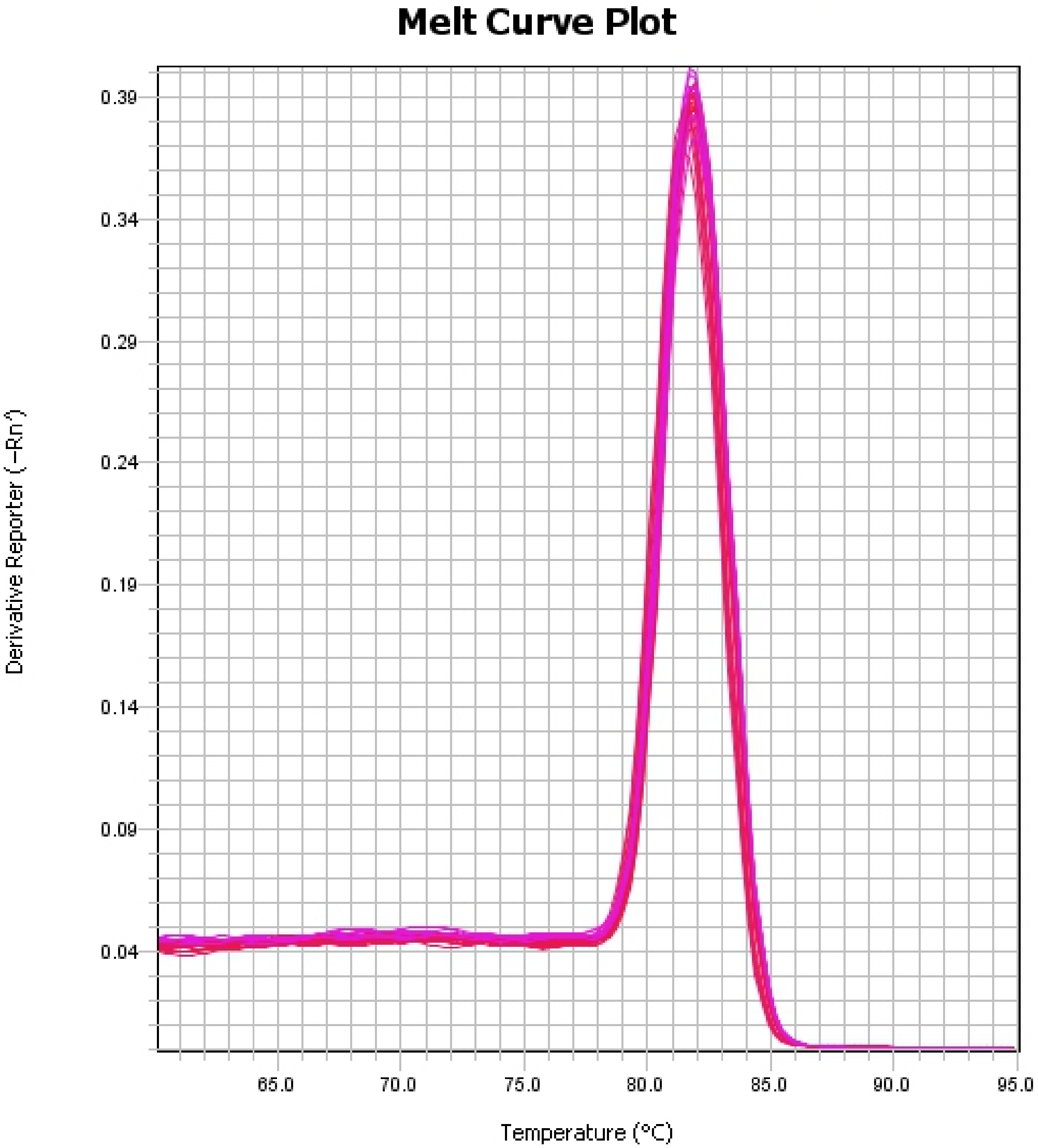

Figure S19 Melt Curve Plot psme4b

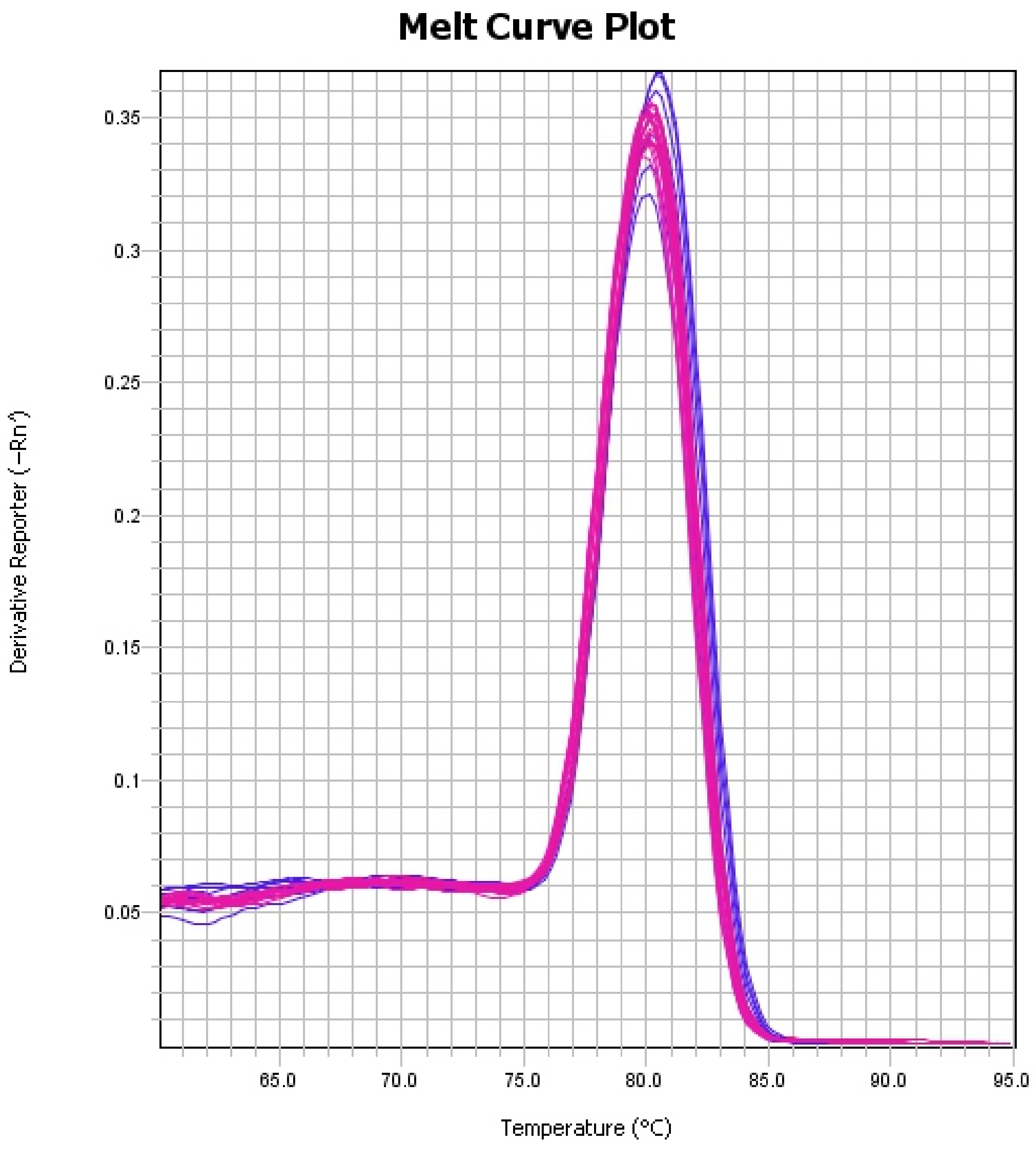

Figure S20 Melt Curve Plot sirp71-45k5.4

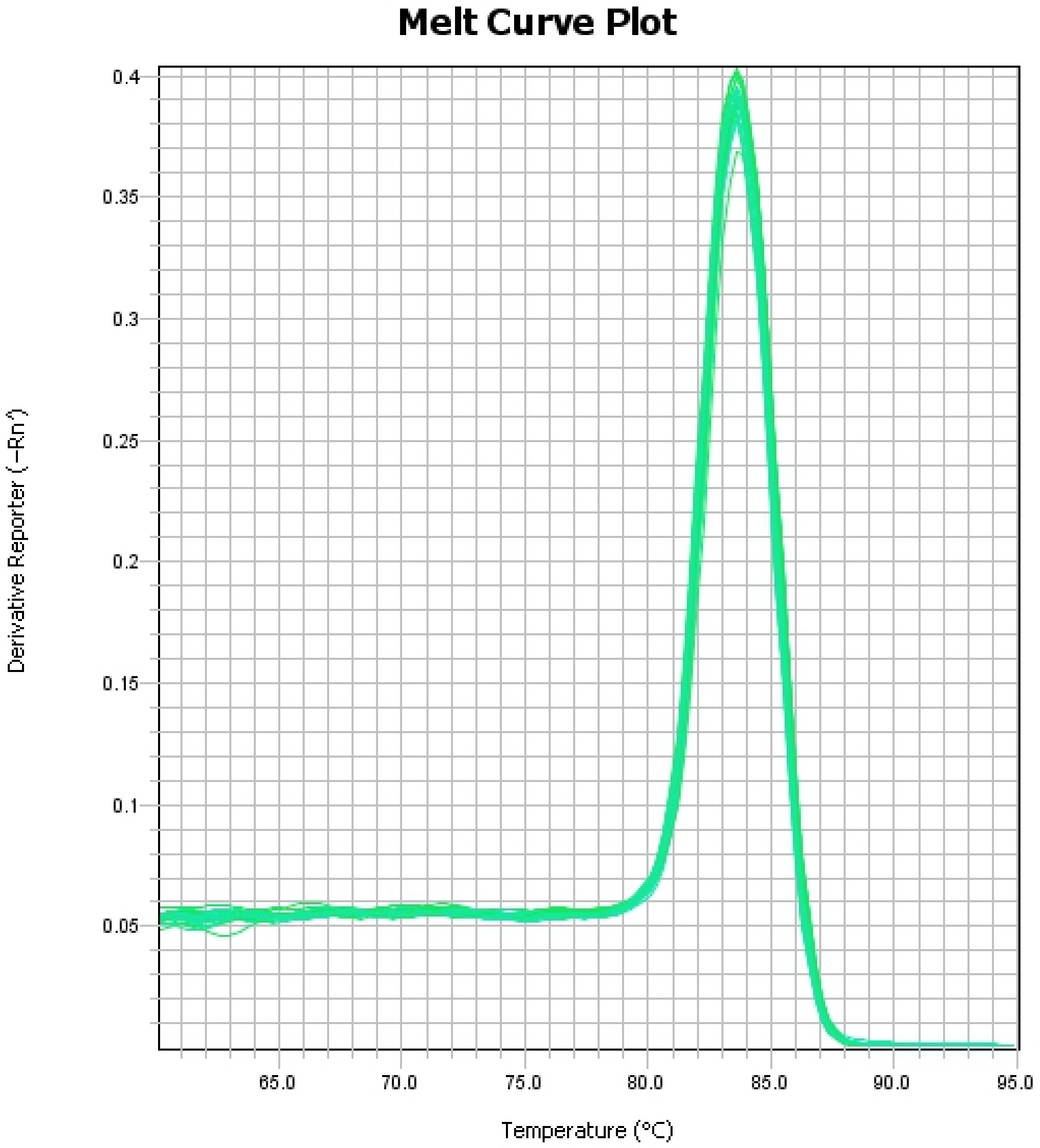

Figure S21 Standard Curve psma3

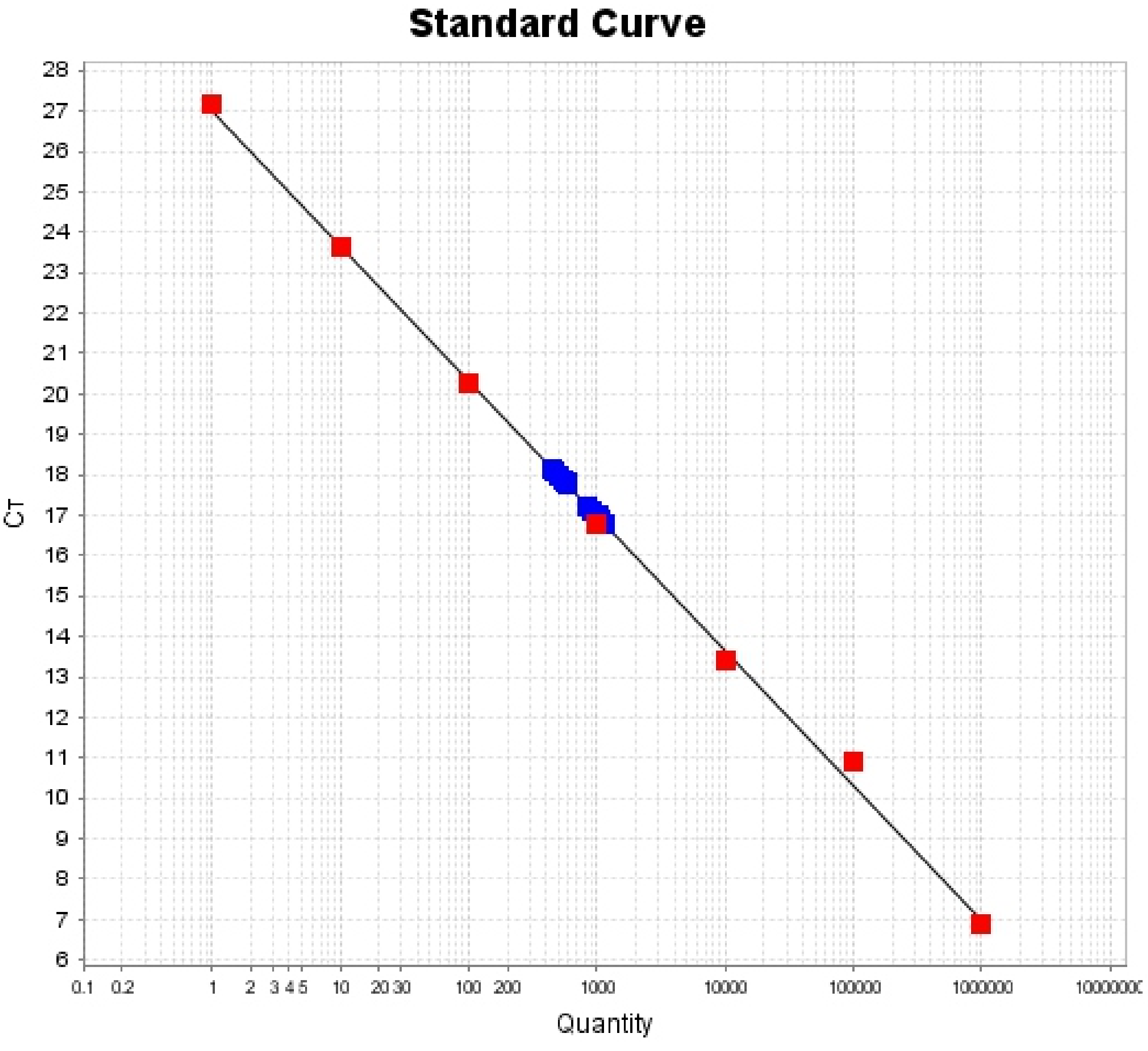

Target: Target 1 Slope: -3.334 Y-Inter: 27.005 R^2: 0.998 Eff%: 99.503 Error: 0.059

Figure S22 Standard Curve psmc2

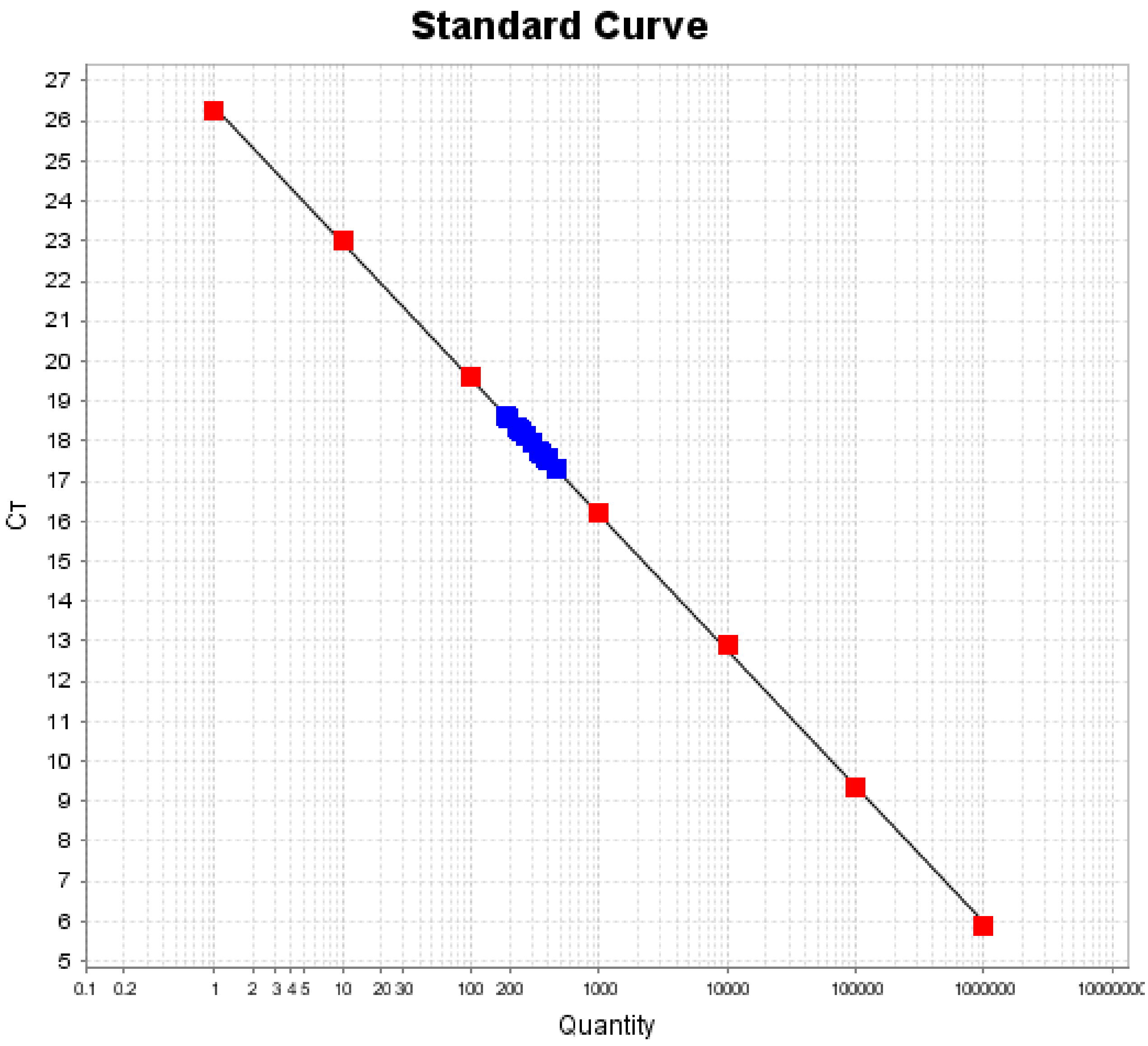

Target: Target 1 Slope: -3.401 Y-Inter: 26.38 R^2: 1 Eff%: 96.807 Error: 0.02

Figure S23 Standard Curve psmc3

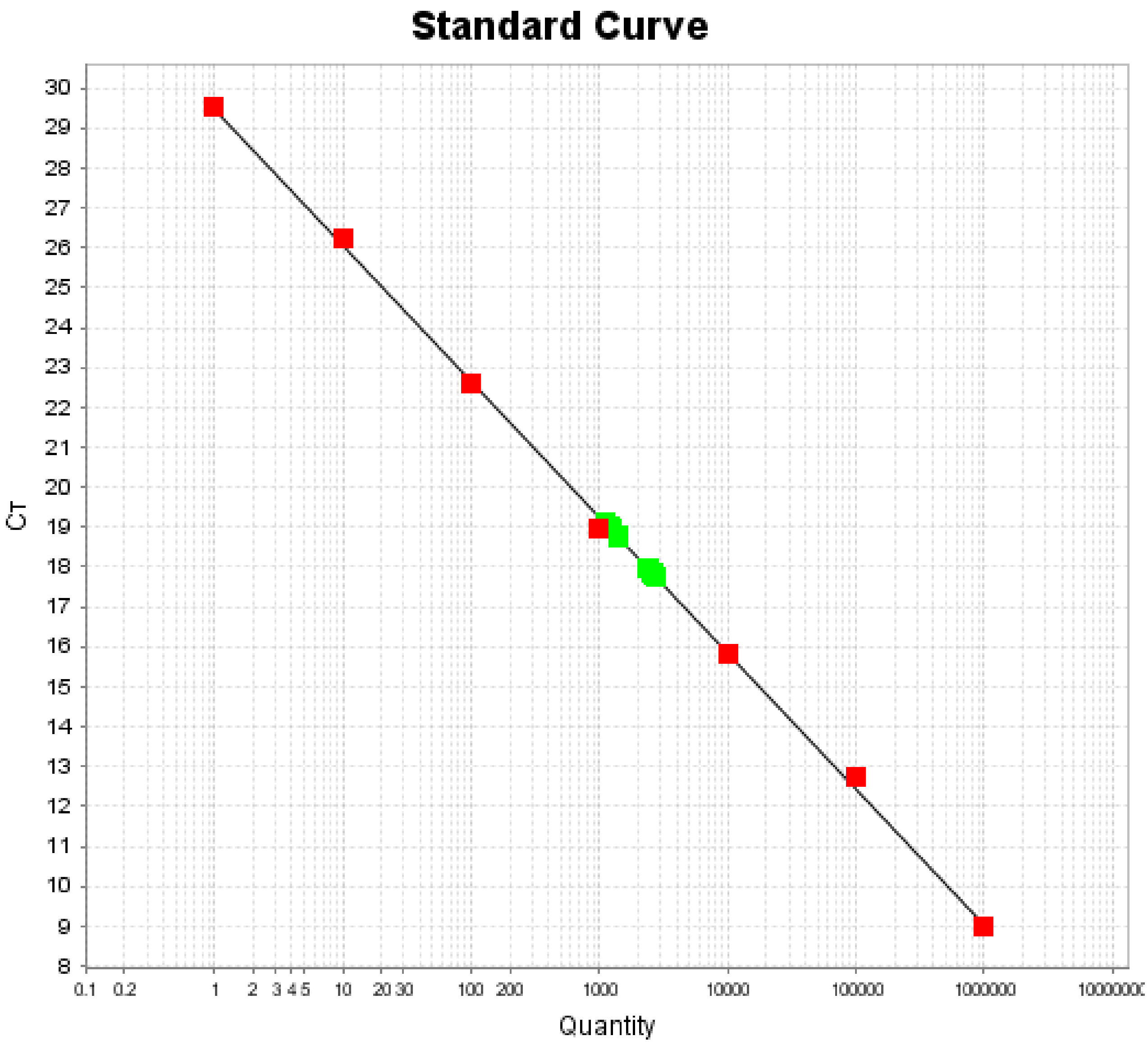

Target: Target 1 Slope: -3.412 Y-Inter: 29.5 R^2: 0.999 Eff%: 96.378 Error: 0.039

Figure S24 Standard Curve psmc6

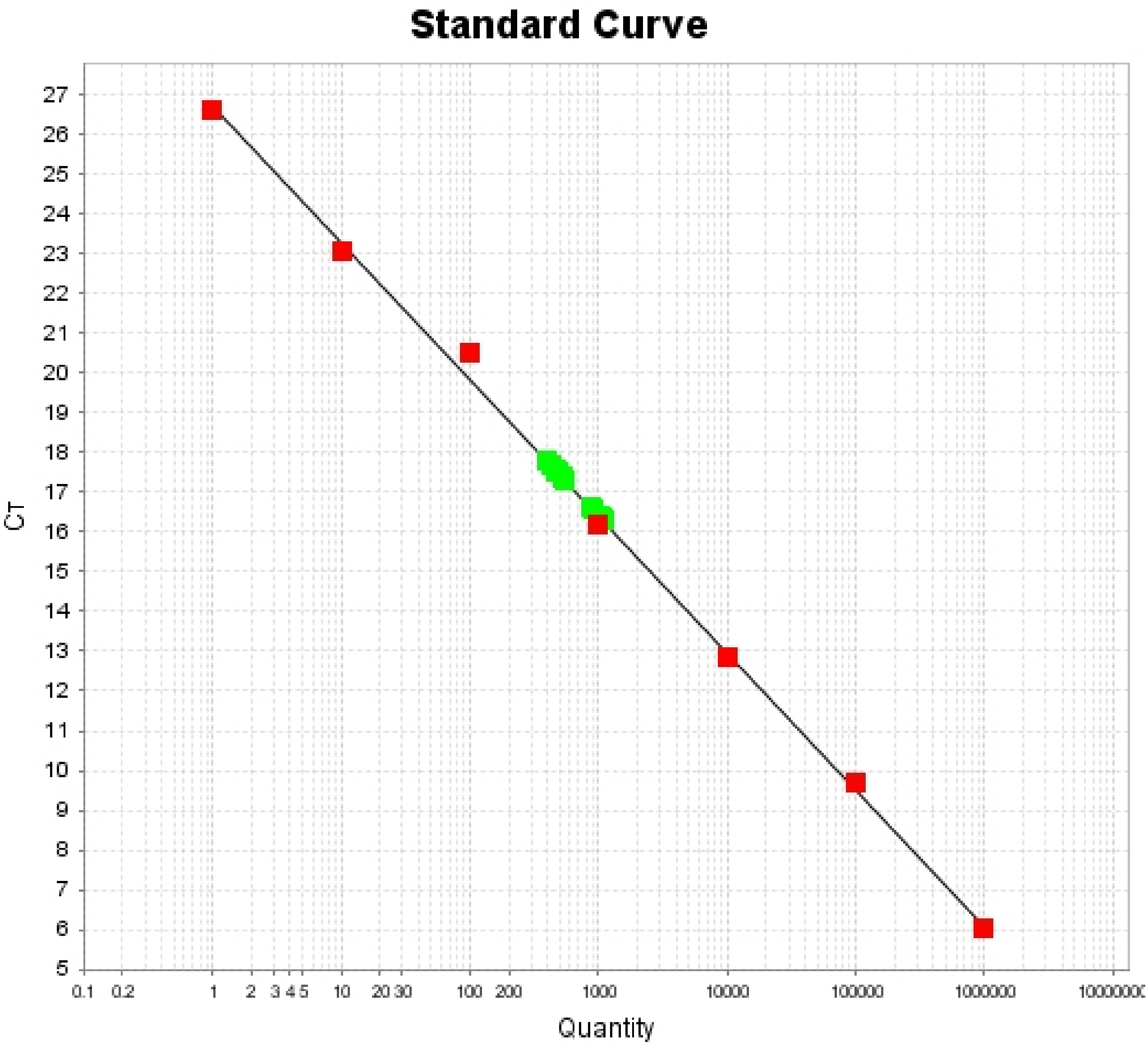

Target: Target 1 Slope: -3.442 Y-Inter: 26.755 R^2: 0.998 Eff%: 95.218 Error: 0.066

Figure S25 Standard Curve psmd4a

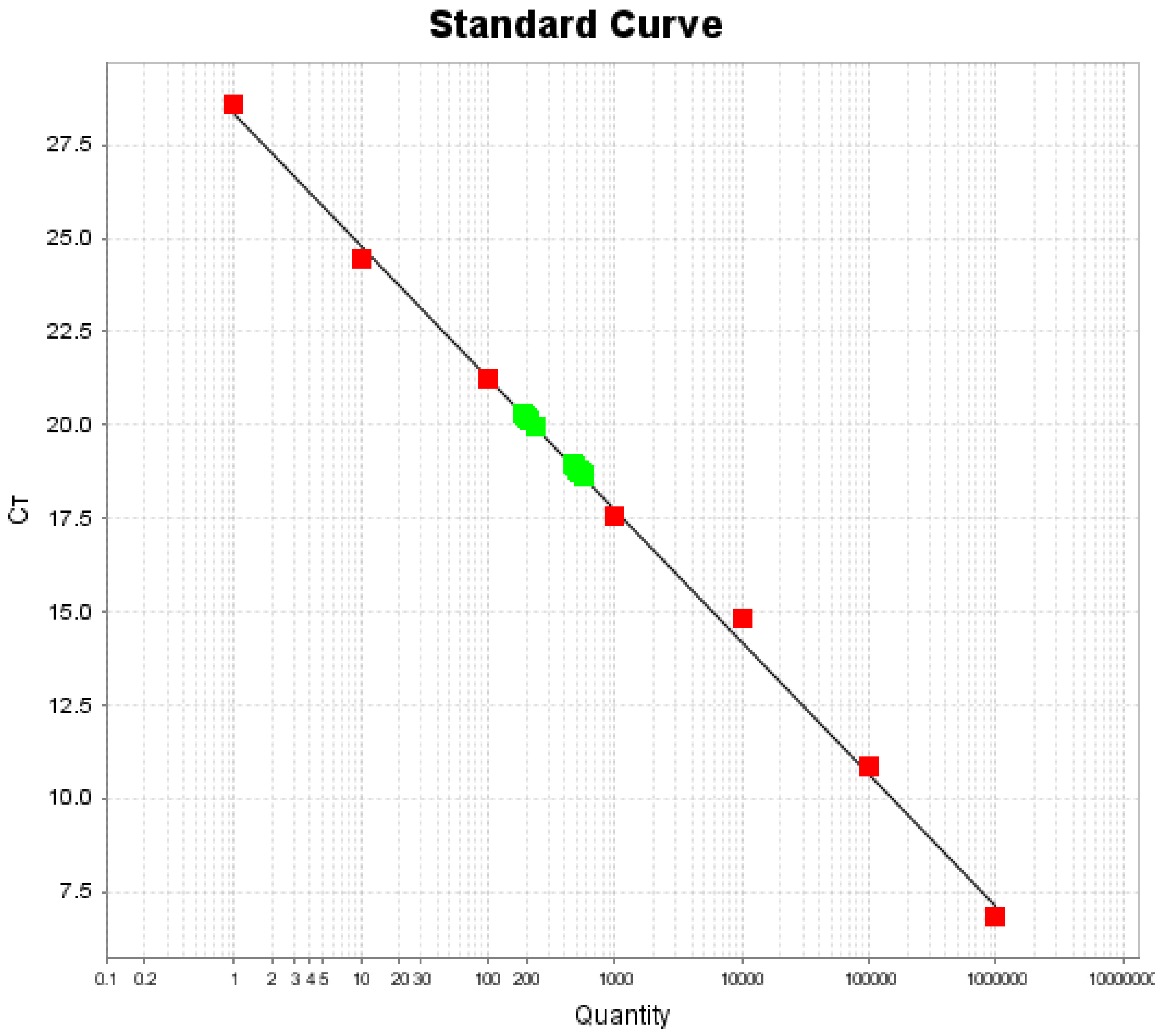

Target: Target 1 Slope: -3.537 Y-Inter: 28.374 R^2: 0.998 Eff%: 91.746 Error: 0.073

Figure S26 Standard Curve psmd8

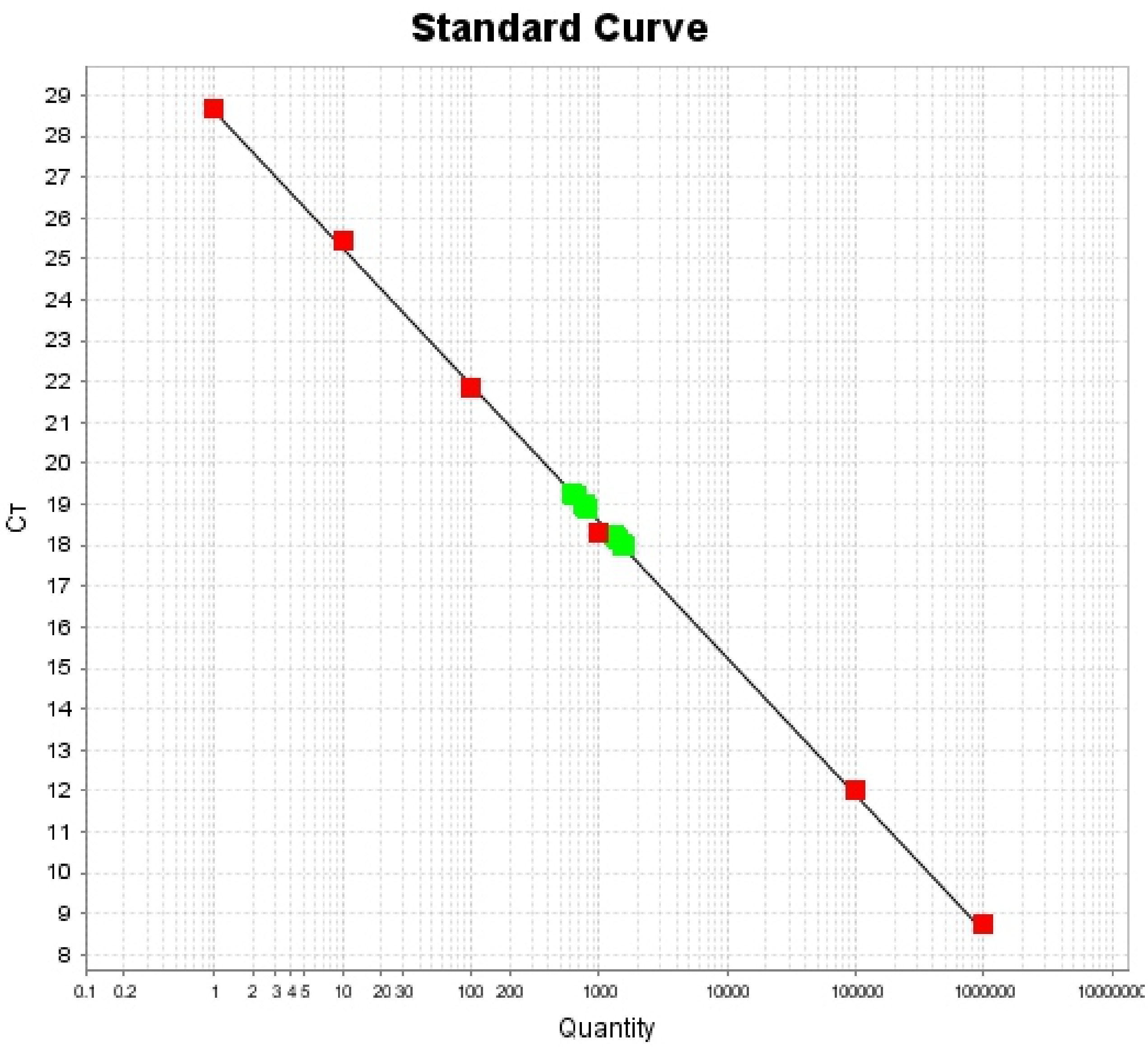

Target: Target 1 Slope: -3.335 Y-Inter: 28.621 R^2: 0.999 Eff%: 99.457 Error: 0.039

Figure S27 Standard Curve psmd11a

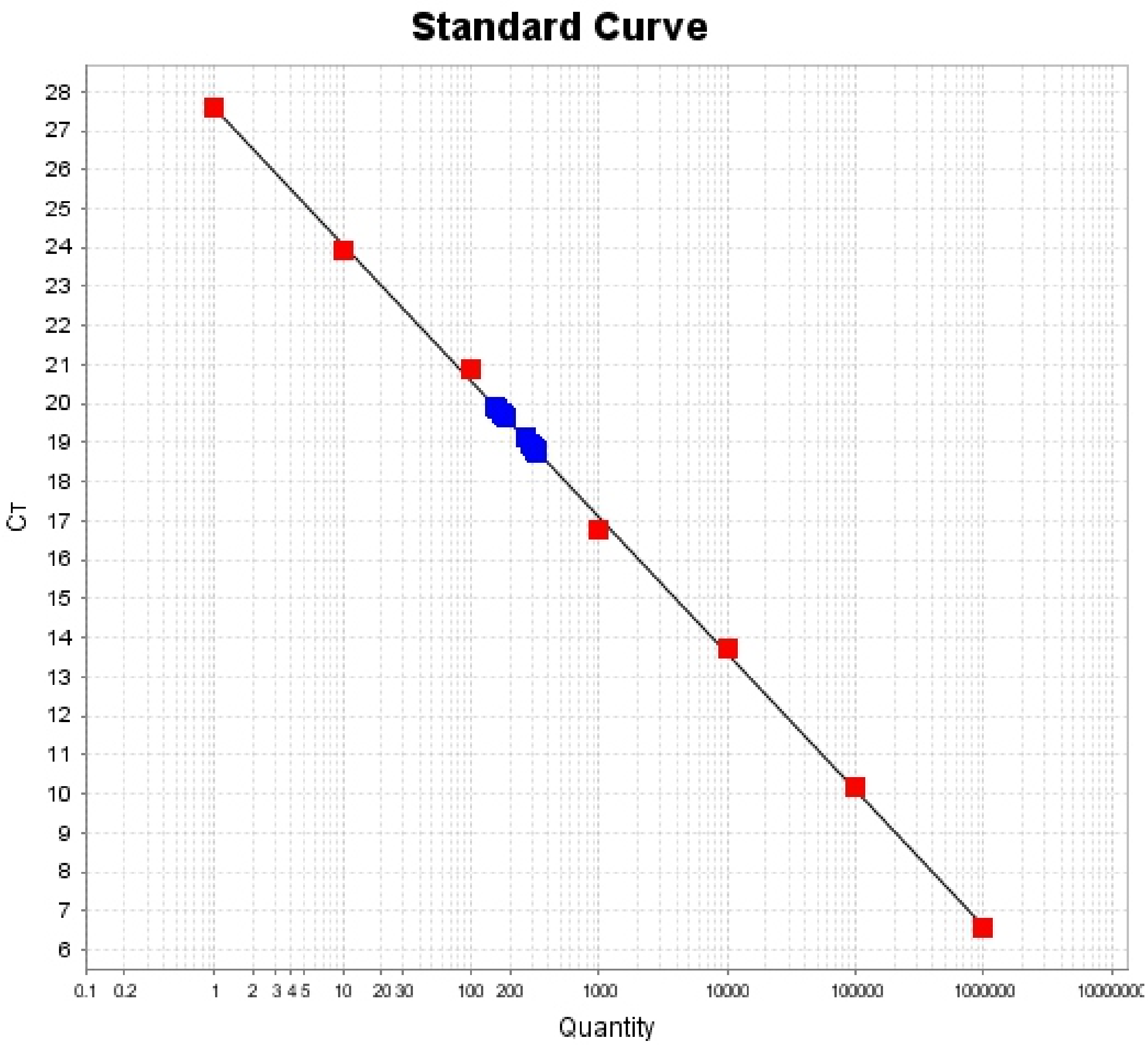

Target: Target 1 Slope: -3.494 Y-Inter: 27.572 R^2: 0.999 Eff%: 93.284 Error: 0.044

Figure S28 Standard Curve psme3

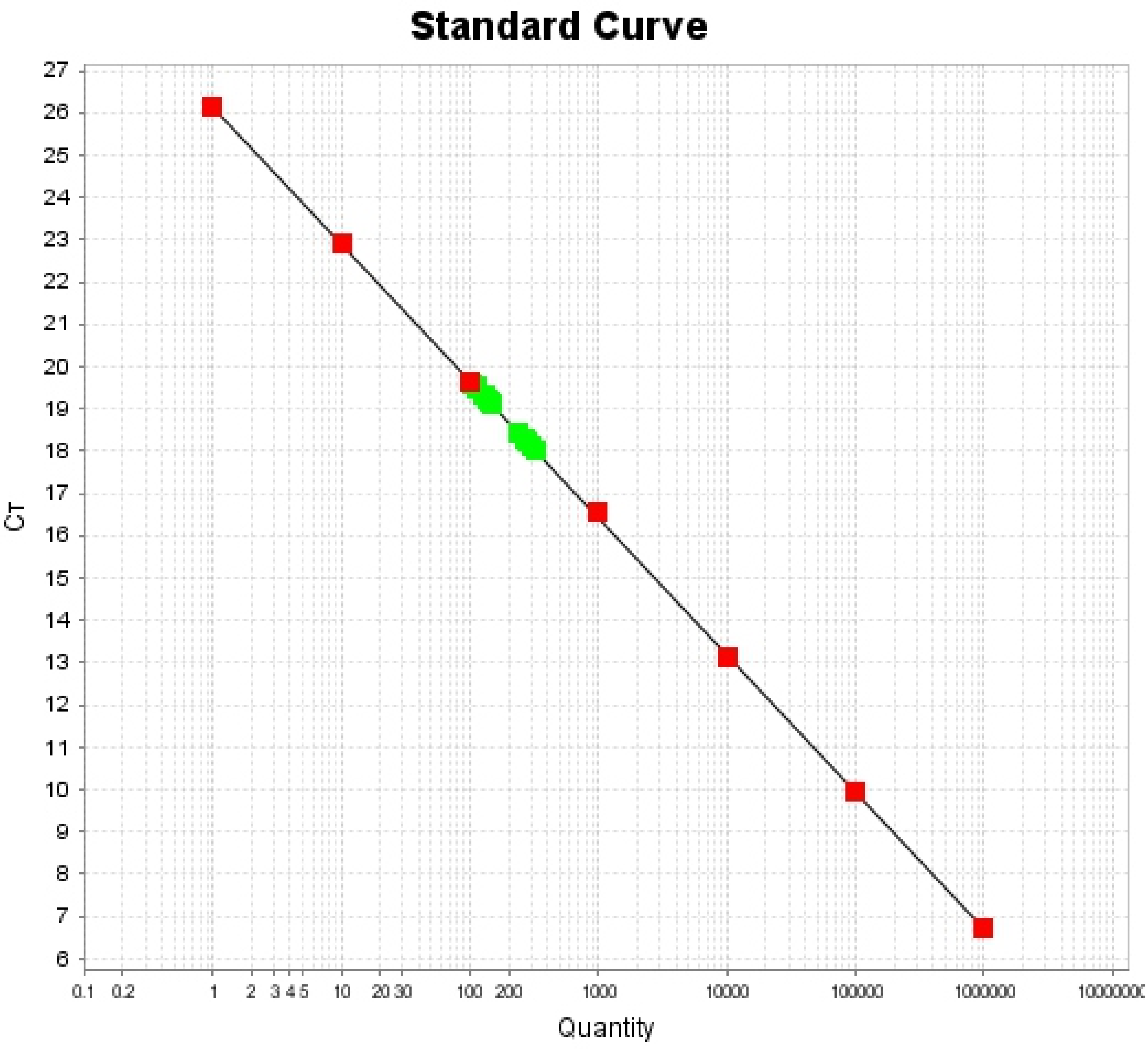

Target: Target 1 Slope: -3.242 Y-Inter: 26.154 R^2: 1 Eff%: 103.438 Error: 0.015

Figure S29 Standard Curve psme4b

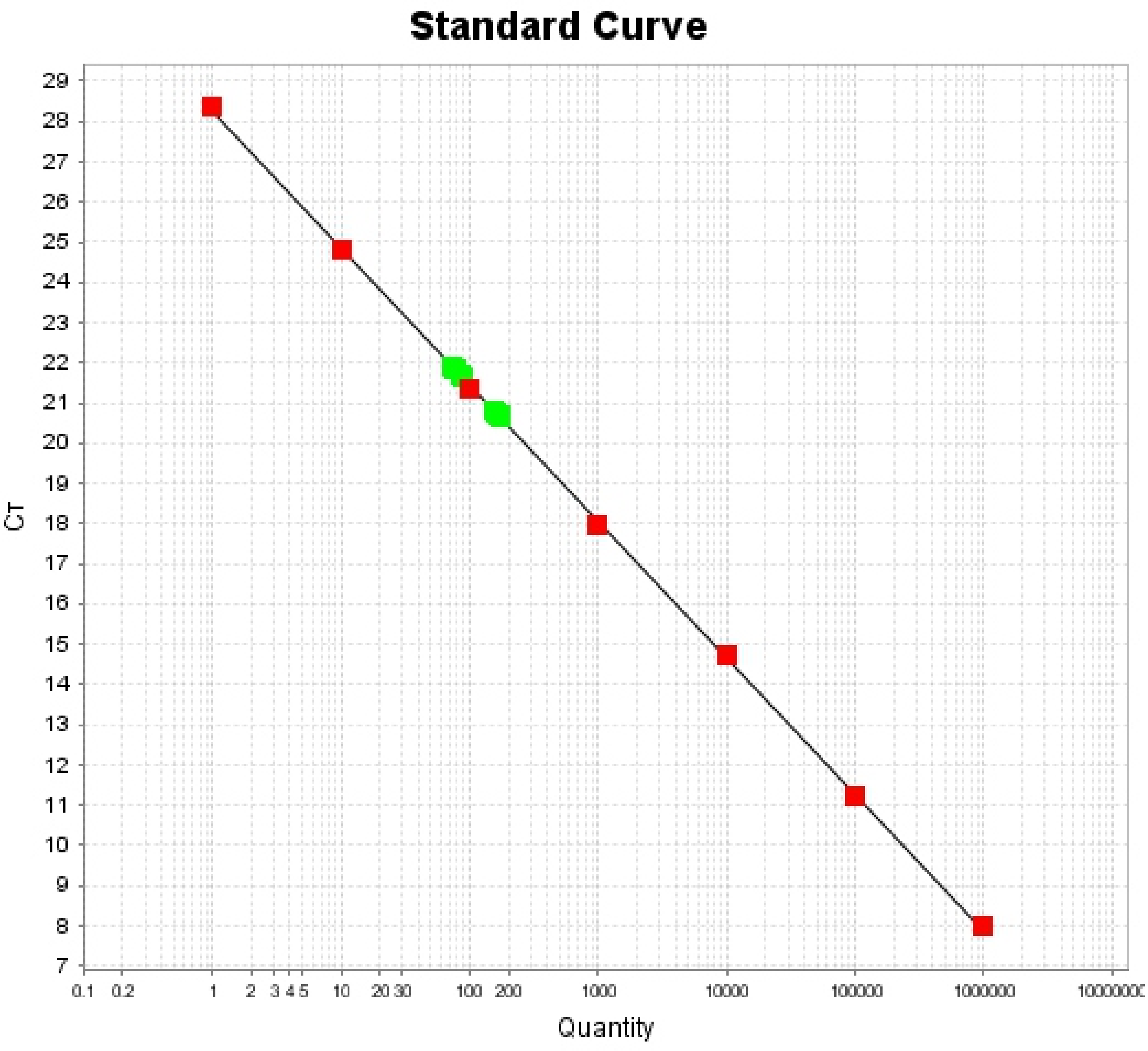

Target: Target 1 Slope: -3.39 Y-Inter: 28.246 R^2: 1 Eff%: 97.236 Error: 0.02

Figure S30 Standard Curve sirp71-45k5.4

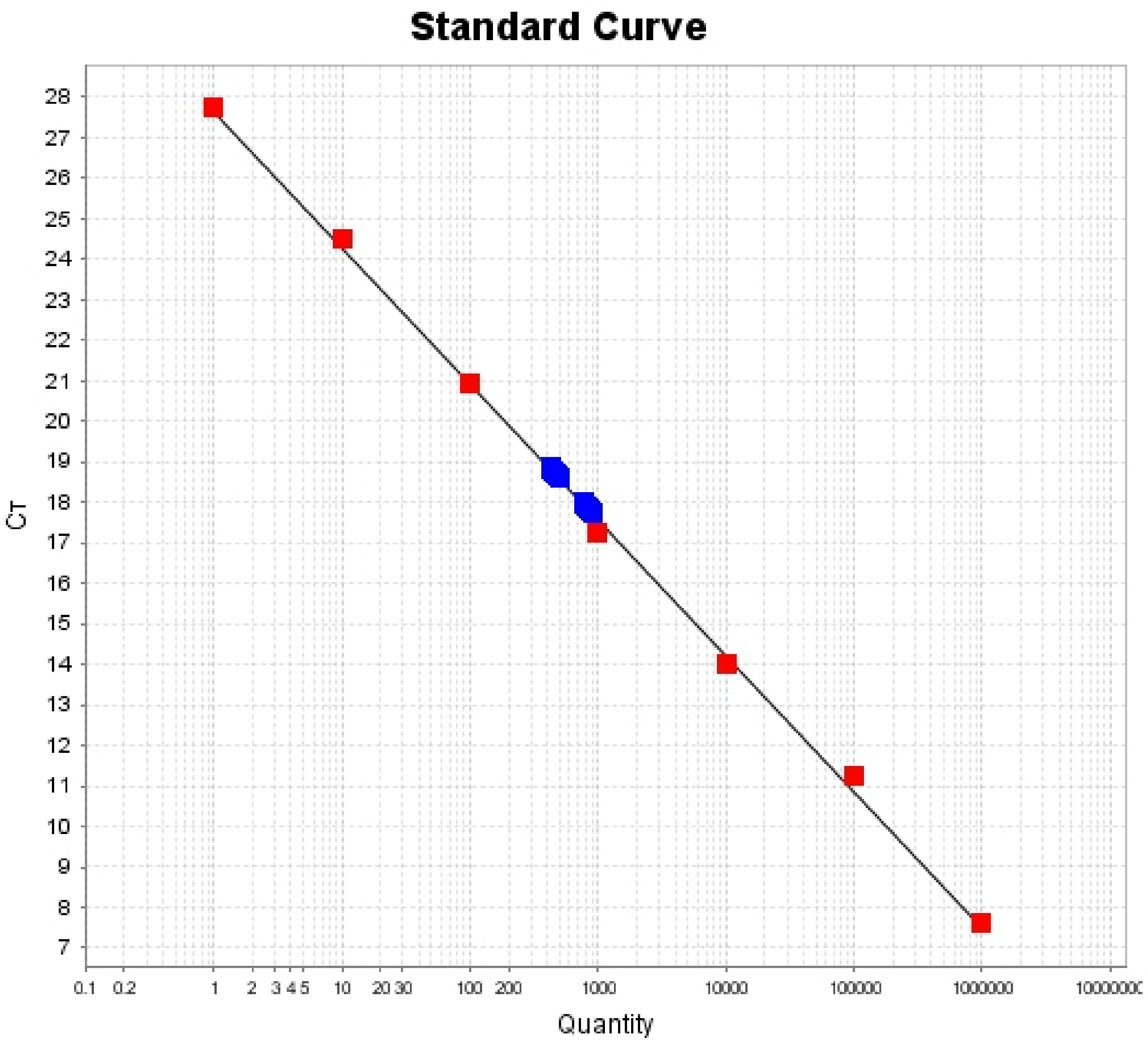

Target: Target 1 Slope: -3.356 Y-Inter: 27.673 R^2: 0.999 Eff%: 98.61 Error: 0.05
